# On the turbulence structure of deep katabatic flows on a gentle mesoscale slope

**DOI:** 10.1002/qj.3734

**Published:** 2020-01-23

**Authors:** Ivana Stiperski, Albert A. M. Holtslag, Manuela Lehner, Sebastian W. Hoch, C. David Whiteman

**Affiliations:** ^1^ Department of Atmospheric and Cryospheric Sciences, University of Innsbruck Innsbruck Austria; ^2^ Meteorology and Air Quality Section Wageningen University Wageningen The Netherlands; ^3^ Department of Atmospheric Sciences, University of Utah Salt Lake City Utah

**Keywords:** boundary‐layer depth, low‐level jet, scaling regimes, stable boundary layer

## Abstract

A comprehensive analysis of the turbulence structure of relatively deep midlatitude katabatic flows (with jet maxima between 20 and 50 m) developing over a gentle (1°) mesoscale slope with a long fetch upstream of the Meteor Crater in Arizona is presented. The turbulence structure of flow below the katabatic jet maximum shows many similarities with the turbulence structure of shallower katabatic flows, with decreasing turbulence fluxes with height and almost constant turbulent Prandtl number. Still stark differences occur above the jet maximum where turbulence is suppressed by strong stability, is anisotropic and there is a large sub‐mesoscale contribution to the flux. Detecting the stable boundary‐layer top depends on the method used (flux‐ vs. anisotropy‐profiles) but both methods are highly correlated. The top of the stable boundary layer, however, mostly deviates from the jet maximum height or the top of the near‐surface inversion. The flat‐terrain formulations for the boundary‐layer height correlate well with the detected top of the stable boundary layer if the near‐surface and not the background stratification is used in their formulations; however, they mostly largely overestimate this boundary‐layer height. The difference from flat‐terrain boundary layers is also shown through the dependence of size of the dominant eddy with height. In katabatic flows the eddy size is semi‐constant with height throughout the stable boundary‐layer depth, whereas in flat terrain, eddy size varies significantly with height. Flux‐gradient and flux‐variance relationships show that turbulence data from different stable boundary‐layer scaling regimes collapse on top of each other showing that the dominant dependence is not on the scaling regime but on the local stability.

## Graphical abstract

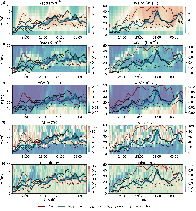

Persistent relatively deep katabatic flows develop over a shallow mesoscale slope outside the Meteor Crater, Arizona. Their turbulent structure deviates from the structure of shallow katabatic flows in that the small‐scale turbulence is strongly attenuated already at heights below the jet maximum. The height of the stable katabatic boundary layer is significantly lower than over flat terrain for the same forcing. The change of turbulence anisotropy below and above the boundary layer provides a new diagnostic of boundary‐layer height.

## 1 INTRODUCTION

Katabatic flows develop when stable boundary layers (SBLs) form over inclined surfaces. In the simplest picture, the flows form due to the downslope component of buoyancy acting to accelerate the flow down the slope against the stably stratified environment and the retarding effects of surface friction, leading to a pronounced low‐level jet. Despite strong near‐surface thermal stratification, the large shear under the jet maximum is a source of continuous turbulence (e.g. Forrer and Rotach, 1997; Monti *et al*., 2002; Grachev *et al*., 2016) so that katabatic boundary layers (BL) are generally turbulent rather than laminar phenomena (cf. Shapiro and Fedorovich, 2008; Fedorovich and Shapiro, 2009).

In midlatitudes, katabatic flows are relatively weak, with low‐level jet maxima found only a few to several tens of metres above ground. In polar latitudes or Antarctica, however, deep katabatic flows can reach hurricane force and control the local climate (e.g. Renfrew, 2004). Lenaerts *et al*. (2017) suggested that katabatic winds could also have an effect on the near‐surface melt, although to a lesser extent than föhn. This near‐surface warming caused by katabatic flows could be due to along‐slope warm‐air advection (Zhong and Whiteman, 2008) as well as due to the near‐surface turbulent mixing caused by the large shear below the katabatic jet maximum that can destabilize the near‐surface layer (van der Avoird and Duynkerke, 1999) and entrain potentially warmer air down to the surface (Pinto *et al*., 2006).

Studies of the mean structure of deeper katabatic flows (e.g. Poulos *et al*., 2000; Monti *et al*., 2002; Renfrew, 2004; Cuxart *et al*., 2007) have shown that the jet maximum height (*h*_jet_) tends to be located in the middle (or the middle third) of the surface‐based inversion. This has important implications for detecting the SBL height in katabatic flows as this height will differ based on whether wind speed, buoyancy or flux profiles are used to infer it (cf. Mahrt *et al*., 1979; Whiteman, 2000; Heinemann, 2004; Shapiro and Fedorovich, 2009). Grisogono and Oerlemans (2001) have shown that the potential cause of this mismatch between *h*_jet_ and inversion height is the variation of eddy diffusivity with height. In simple models, such as the classical Prandtl model (e.g. Egger, 1990) and simple momentum budget approaches (e.g. Oldroyd *et al*., 2014) this variability is not taken into account so the jet maximum occurs at the top of the inversion where the buoyancy term driving the flow becomes zero. Some observations (e.g. Oldroyd *et al*., 2014; Grachev *et al*., 2016) suggest that this is often the case for shallow flows.

While there are numerous studies of the mean flow characteristics of katabatic flows (see, e.g., overviews by Zardi and Whiteman (2012), Grachev *et al*. (2016) and Jensen *et al*. (2017)), turbulence studies are scarce, especially for deeper flows in which the jet maximum is located several tens of metres above ground. The existing observational studies (e.g. Forrer and Rotach, 1997; Denby and Smeets, 2000; Heinemann, 2004; Grachev *et al*., 2016) suggest that the *h*_jet_ imposes a strong control on the turbulent structure of the katabatic flow. At the jet maximum, the streamwise momentum flux and the streamwise heat flux change their sign, while the turbulence kinetic energy (TKE) and its dissipation rate *ϵ* have a minimum, and potential temperature variance an absolute maximum (cf. Denby, 1999; Grachev *et al*., 2016).

The importance of *h*_jet_ and the fact that turbulent fluxes in katabatic flows vary strongly with height, even very close to the surface (Parmhed *et al*., 2004; Nadeau *et al*., 2013; Oldroyd *et al*., 2014), suggest that flat‐terrain surface‐layer scaling is not the appropriate scaling framework for katabatic flows. Employing a local scaling framework that allows the variation of fluxes with height, Forrer and Rotach (1997), Nadeau *et al*. (2013) and Grachev *et al*. (2016) showed that turbulence data do collapse onto scaling curves, but the scaling curves themselves differ between the studies. Data above *h*_jet_ followed *z*‐less scaling in the case of Grachev *et al*. (2016), but showed an increase of scaled variances with stability in the study of Nadeau *et al*. (2013). On the other hand, Denby and Smeets (2000) and Heinemann (2004) suggested that the local scaling framework is also not suitable for katabatic flows as alternative length‐scales (*h*_jet_ and buoyancy length‐scale) are more relevant than the surface Obukhov length *L* in governing the turbulence dynamics, and suggested that their use as scaling variables should improve the collapse of data onto a single scaling curve. The scaling framework of Denby and Smeets (2000) using *h*_jet_ was indeed comparatively successful for scaled momentum flux but their non‐dimensional shear showed large scatter.

Whether the turbulent structure in deeper katabatic flows differs from their shallow counterparts and resembles more the SBL structure over flat terrain (Van der Avoird and Duynkerke, 1999) may thus depend on the relation between *h*_jet_, as the height where momentum flux and TKE approach zero, and the surface Obukhov length *L* (cf. Parmhed *et al*., 2004; Grisogono *et al*., 2007). Indeed, for shallow katabatic flows *h*_jet_ is generally smaller than *L* and could therefore be considered as the relevant length scale for turbulent mixing (e.g. Smeets *et al*., 1998; Denby and Smeets, 2000; Axelsen and van Dop, 2009; Oldroyd *et al*., 2014; Grachev *et al*., 2016). However, deeper katabatic flows with jet maxima at heights greater than the *L* have been predicted to develop a canonical surface layer, that is, one in which turbulence is governed by surface‐related scales (cf. Grisogono *et al*., 2007) and fluxes are quasi‐constant with height. The depth of the surface layer, commonly assumed to be 10% of the total SBL depth (Stull, 1988), however, might be well below the lowest measurement level (cf. Grisogono and Oerlemans, 2001).

The Second Meteor Crater Experiment (METCRAX II: Lehner *et al*., 2016) provides a unique dataset of high‐resolution turbulence measurements of persistent relatively deep katabatic flows that can be used to answer some of the questions raised above. Developing over a gentle and extensive mesoscale slope outside Arizona's Meteor Crater, these katabatic flows control the complex flow structure within the crater which was the focus of the METCRAX II campaign (Adler *et al*., 2012; Lehner *et al*., 2016; Whiteman *et al*., 2018a; 2018b; Lehner *et al*., 2019). We focus our study on multiple intensive observation periods (IOPs) with well‐developed katabatic flows and a night with synoptically induced flow with the aim of answering the following questions:

1. How does the turbulence structure of relatively deep katabatic flows differ from their more commonly studied shallow katabatic counterparts?
2. What is the dominant length‐scale governing the turbulence structure of these katabatic flows?
3. How does this length‐scale relate to *h*_jet_, the surface Obukhov length *L* and, given the low slope angle, to the SBL height over flat terrain?
4. Is a canonical surface layer with constant fluxes and obeying surface‐layer scaling able to develop in these deeper katabatic flows?

Here we use the lowest measurement level to define the surface Obukhov length, despite the fact that in cases when no surface layer can be detected this value will differ from the true surface value.

This article is organized as follows. In Section 2 we give an overview of the dataset and data analysis. Section 3 focuses on the mean flow and turbulence characteristics of deeper katabatic flows, contrasting them to the shallower flows. Section 4 examines different ways of detecting the height of the katabatic SBL and tests different theoretical formulations for detecting the SBL height. Section 5 examines SBL scaling regimes for katabatic flow, while Section 6 discusses the key results and presents conclusions.

## 2 METHODOLOGY

### 2.1 Observational sites and instrumentation

The METCRAX II campaign took place during October 2013 at the Barringer Meteorite Crater (a.k.a. Meteor Crater) in Arizona. The main meteorological and turbulence data analysed here were collected by the National Center for Atmospheric Research (NCAR) Integrated Surface Flux System (UCAR/NCAR, 1990; Sun *et al*., 2003). A description of the sensors, sensor accuracy, calibration, and planar fit post‐processing procedure can be found at the NCAR METCRAX II website (www.eol.ucar.edu/content/isfs‐metcraxii).

**Figure 1:**
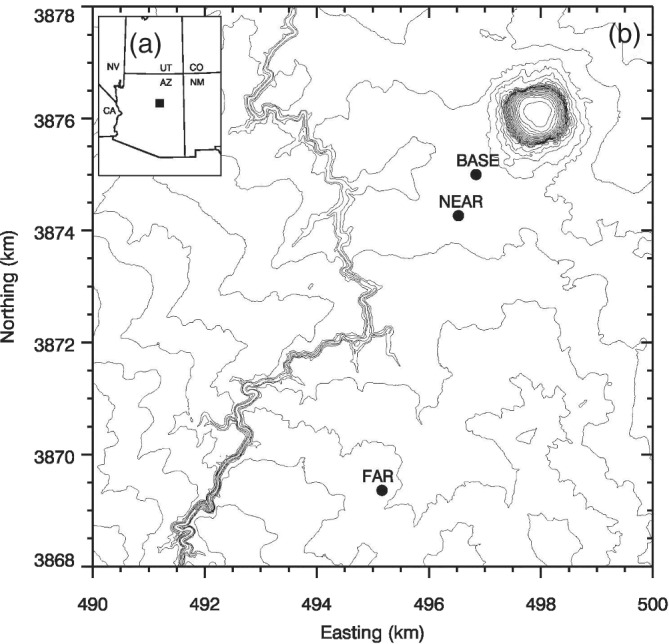
(a–b) Topographic map (Universal Transverse Mercator 12S projection, 10 m elevation contours) of the area upwind of the Arizona Meteor Crater. NEAR tower (1,687 m ASL), FAR site (1,724 m ASL), and BASE radiosonde base station (1,695 m ASL) locations are indicated

The main data come from a heavily instrumented 50 m tower on the sloping Colorado Plateau 40 km east of Flagstaff, Arizona. This 50 m flux tower called NEAR was located on a slightly rolling plain that is sparsely covered with shrubs and desert grasses and slopes gently upward (∼1° slope) on the regional (∼30 km) scale toward the southwest (average orientation of the mesoscale slope is 215°). The tower was located 1.8 km southwest of the Meteor Crater at a location that was not affected by upstream blocking from the crater's elevated rim (Figure 1). The NEAR tower was instrumented at 10 levels with Campbell CSAT3 sonic anemometers and aspirated hygrothermometers, with the lowest level located at 3 m, followed by nine levels every 5 m from 10 to 50 m. A microbarometer (PTB220) collected pressure data at the 2 m level. Turbulence data were also collected with a CSAT3 sonic anemometer at the 3 m level of the 10 m FAR tower located 5 km south‐southwest of the NEAR tower (Figure 1). All turbulence data were measured at a frequency of 20 Hz. Additionally, during IOPs tethered‐balloon soundings were taken at 20 min intervals at the BASE site 0.8 km north‐northeast of the NEAR tower (Figure 1).

### 2.2 Selection of study periods

Data from six southwesterly katabatic flow events during IOPs 1, 2, 3, 4, 6 and 7 (cf. Lehner *et al*., 2016) are the main focus of this study. These IOPs were characterized by weak synoptic forcing and mostly clear sky conditions (the exceptions are IOP 6 which was mostly overcast with cirrus and altocumulus cloud cover, and IOP 7 with a cirrus cloud cover) during which persistent katabatic flows developed in the METCRAX domain. Whereas IOPs 1, 2, 6 and 7 were characterized by a shallower jet (maximum at ∼20 m), IOPs 3 and 4 had a deeper jet (maximum at ∼40 m). The occurrence of shallower and deeper jets was already recognized during the METCRAX I campaign (Savage *et al*., 2008).

Within each IOP we define the katabatic flow periods as night‐time periods (1930 to 0400 MST (UTC – 7 hr)) when the wind direction at the lowest tower level was from the wind sector 150 to 250° and the atmosphere was stable (*z/*Λ > 0) at 3 m. Here *z* is the height above ground and Λ the local Obukhov length. This definition intentionally eliminates evening‐transition periods, as our interest is the turbulence structure of well‐developed katabatic flows. We did not require that data are stable at all levels because counter‐gradient positive sensible‐heat fluxes commonly occurred at higher levels of the tower (more than 10% of the time). A special observation period (SOP) on the night of 28–29 October 2013, with a strong synoptically forced windstorm during which katabatic jet maximum was not detectable, is used for comparison to the katabatic flow periods.

### 2.3 Turbulence analysis

A one‐minute averaging time was used to define turbulence fluctuations. Such a short averaging time was used to avoid contamination of fluxes and other turbulence statistics by non‐turbulent (sub)mesoscale motions and was based on the results of multi‐resolution flux decomposition (MRD: e.g. Howell and Mahrt, 1997; Vickers and Mahrt, 2003; cf. Figure 6). To reduce the random error, however, the one‐minute block‐averaged turbulence statistics were subsequently averaged to 5 min, and will be used in the rest of this study. In addition, we calculated the anisotropy at a 5 min averaging scale. This scale is close to the gap scale for horizontal velocity variances at measurement levels close to the surface but it already contains contributions from submesoscale motions at higher levels.

**Figure 2:**
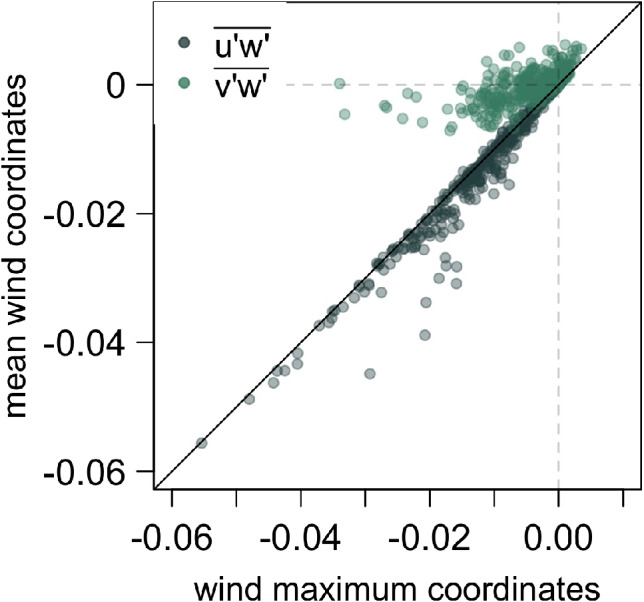
Horizontal momentum fluxes in the coordinate system defined by the wind direction at the jet maximum (*x‐*axis) and the natural coordinate system defined by the mean wind direction at each height (*y*‐axis). Shown are all vertical levels for all IOPs except IOP5 [Colour figure can be viewed at wileyonlinelibrary.com]

The coordinate system we use is defined as follows: data were first tilt‐corrected by applying the planar fit method (Wilczak *et al*., 2001), followed by a coordinate rotation at each tower level into the wind direction at the jet maximum. In this coordinate system we define the *u* component of velocity as streamwise velocity, *v* as spanwise velocity and *w* as slope‐normal velocity. Due to the low slope angle, however, the difference between true vertical and slope normal is negligible (0.01%). We chose this coordinate system under the assumption that this is the direction of the dominant katabatic forcing, although it might deviate from the local downslope direction due to small‐scale local inhomogeneities or the orientation of the mesoscale pressure gradient. This choice of coordinate system allows for a most straightforward investigation of budgets as the buoyancy terms are the only terms affected by the change of wind direction with time in as much as this direction does not coincide with the dominant downslope direction. This coordinate system does have an impact on the magnitude of the streamwise momentum and heat fluxes at the surface where these are the largest, and the wind directional change is relevant (Figure 2). The largest effect, though, is seen for the *v*‐component of the momentum flux. Above *h*_jet_ where the wind directional change is the largest, the flux values (all except streamwise heat flux) are small so that the effect of the coordinate system is negligible. The choice of the coordinate system, however, does not impact the conclusions drawn from the results.

Wind speed and potential temperature gradients were calculated analytically by fitting functions of the following types to the wind speed and potential temperature profiles:

(1) $$f_{1}\left(z\right)=a+\text{bz}+cz^{2}+d\ln z,$$

(2) $$f_{2}\left(z\right)=a+\text{bz}+cz^{2}+d\ln z+e\ln z^{2},$$

(3) $$f_{3}\left(z\right)=a+\text{bz}+cz^{2}+d\ln z+e\ln z^{2}+f\ln z^{3}.$$

Two analytical functions were used for each variable, *f*_1_(*z*) and *f*_2_(*z*) for the temperature profiles, and *f*_2_(*z*) and *f*_3_(*z*) for the wind speed profiles. The performance of the two interpolation schemes was evaluated at each time step by calculating the root‐mean‐square error. The scheme with the smaller root‐mean‐square error (RMSE) was subsequently chosen for that averaging period. The high degree of the polynomial fit was necessary to correctly reproduce the shape of the low‐level jet; however, it sometimes caused unphysical curvature of the fitted profile at the lowest two measurement levels. For this reason, a log‐linear fit was calculated between the surface (roughness length was calculated from the neutral profiles to be 0.053 m) and the three lowest measurement levels. This log‐linear fit provided the gradients for the 3 and 10 m levels. For cases where even the log‐linear fit provided an erroneous negative gradient at the first level even though the wind maximum was above that level, we used finite differences. Finally, *h*_jet_ was calculated as the height where the fitted wind speed profiles had a maximum. This estimate was deemed better than determining the maximum from the actual measurements due to the 5 m distance between the measurement heights.

The fitted profiles allowed an analytical calculation of wind speed and potential temperature gradients as well as gradient (*R*_*i*_) and flux (*R*_*f*_) Richardson numbers, defined as:

(4) $$R_{i}=\frac{g}{\theta_{0}}\;\frac{\frac{\partial \theta }{\partial z}}{{\left(\frac{\partial U}{\partial z}\right)}^{2}+{\left(\frac{\partial V}{\partial z}\right)}^{2}},$$

(5) $$R_{f}=\frac{g}{\theta_{0}}\;\frac{\bar{w^{\prime }\theta^{\prime }}\cdot \cos\alpha -\bar{u^{\prime }\theta^{\prime }}\cdot \sin\alpha_{u}-\bar{v^{\prime }\theta^{\prime }}\cdot \sin\alpha_{v}}{\bar{u^{\prime }w^{\prime }}\frac{\partial U}{\partial z}+\bar{v^{\prime }w^{\prime }}\frac{\partial V}{\partial z}}\;.$$

Here *θ*_0_ is the mean potential temperature in the layer below each measurement height, and the two angles *α*_*u*_ and *α*_*v*_ in *R*_*f*_ are functions of the slope angle *α* (1°) and account for the fact that the wind direction at the jet maximum might not coincide perfectly with the orientation of the slope. Following Oldroyd *et al*. (2016), they are defined as:

(6) $$\alpha_{u}=\arcsin\left(\cos\psi \sin\alpha \right),$$

(7) $$\alpha_{v}=\arcsin\left(\cos\left(\psi -90\right)\sin\alpha \right).$$

Here *ψ* is the difference between the average slope orientation of 215° and the wind direction at the wind maximum that defines the coordinate system.

Finally, we define the turbulent Prandtl number as:

(8) $$P_{r}=\frac{R_{i}}{R_{f}}.$$

The terms of the momentum, TKE and flux budgets were calculated using forward finite differences due to issues associated with fitting analytical profiles through turbulence quantities, particularly the higher‐order statistics, such as TKE transport, which do not have a known analytical form (cf. Freire *et al*., 2019a). Due to ambiguity of estimating the TKE dissipation rate *ϵ* from sonic data (cf. Chamecki and Dias, 2004), we tested two common methods: the inertial dissipation method (cf. Piper and Lundquist, 2004) where *ϵ* is estimated from the inertial sub‐range of the streamwise velocity power spectrum and the method using the second‐order structure function (cf. Chamecki and Dias, 2004). Dissipation was only estimated for the part of the power spectrum where the spectral slope was −5/3 and the second‐order structure function had a 2/3 slope, where both slopes were allowed to deviate by 10%. Since the dissipation rates from the second‐order structure function were found to be larger than those from the spectral method, and were better correlated to the sum of the remaining TKE budget terms (correlation coefficient equal to 0.9 for spectral and 0.96 for structure function), in the following we will use *ϵ* estimated from the structure function. This *ϵ* was not corrected for path‐averaging effects (cf. Freire *et al*., 2019b).

MRD spectra and co‐spectra were calculated over a two‐hour moving window, moved by 10 min to obtain more reliable statistics for scale analysis (cf. Figure 6), and over a 30 min window for detecting the MRD length‐scale (cf. Figure 9). The signal was linearly de‐trended prior to the spectral calculation. Taylor's hypothesis was used to calculate the MRD length‐scales from the MRD time‐scales by using the mean wind speed in the corresponding 30 min averaging period.

Free atmospheric stability in the form of buoyancy frequency (*N*) above the inversion layer (*N*_free_) was calculated from tethered balloon soundings. The mean potential temperature gradient was calculated by robust linear regression through the measurement points between 100 and 220 m height. The time series of the obtained 20 min mean gradients was then smoothed using a five‐point moving average filter and subsequently interpolated to the same time‐grid as the other measurements. The buoyancy frequencies from the tower measurements (*N*_low_ and *N*_max_) were calculated from the analytical fit to the potential temperature profiles (Equations 1 and 2) obtained from slow‐response temperature measurements.

Anisotropy analysis was performed following Stiperski and Calaf (2018) by calculating the invariants of the anisotropy stress tensor. Here we use the invariant *y*_B_ defining the barycentric Lumley triangle (Banerjee *et al*., 2007) as a measure of how far a given turbulence state is from the isotropic limit. Larger *y*_B_ indicates turbulence closer to isotropic, while *y*_B_ closer to zero indicates highly anisotropic states.

### 2.4 Data quality and scaling

Data were required to satisfy basic quality requirements, namely satisfying the physical limits. In addition, data used to test the SBL scaling regimes were required to satisfy the stationarity criterion following Foken and Wichura (1996), with data below the standard cut‐off value of 30% considered stationary. Finally, we test the effect of the criterion that the *R*_*f*_ should not exceed 0.21 (cf. Grachev *et al*., 2013; Babić *et al*., 2016) on turbulence scaling relations.

## 3 TURBULENCE CHARACTERISTICS OF KATABATIC FLOWS

### 3.1 Mean turbulence profiles

**Figure 3:**
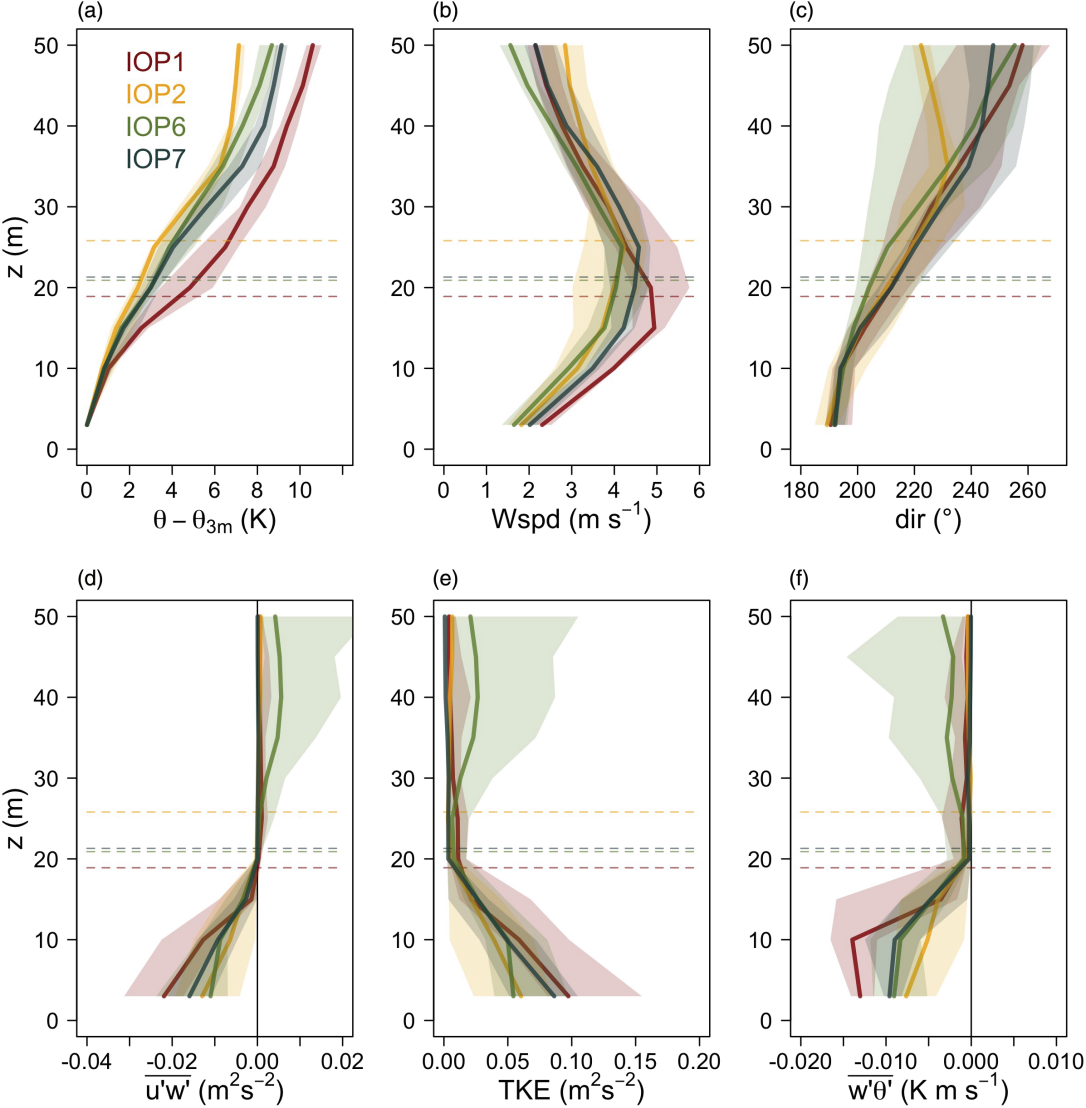
Median profiles of (a) reduced potential temperature (potential temperature minus the value from the lowest level), (b) mean wind speed, (c) wind direction, (d) streamwise momentum flux in the direction of the wind at the jet maximum, (e) turbulence kinetic energy, and (f) slope‐normal kinematic sensible‐heat flux for IOPs 1, 2, 6 and 7 calculated between 2200 and 0000 MST. Shading corresponds to the interquartile ranges. Horizontal dashed lines represent the medians of the jet maximum height (*h*_jet_) for each IOP calculated from the fitted profiles. *h*_jet_ for IOPs 1 and 6 coincide with the those of IOP7 [Colour figure can be viewed at wileyonlinelibrary.com]

We first focus on the general turbulence structure of the katabatic flows developing over the mesoscale slope outside the Meteor Crater and compare this with the turbulence structure of shallow katabatic flows as reported in the literature (i.e. Smeets *et al*., 1998; Grachev *et al*., 2016). Figure 3 shows median profiles for the well‐developed phases of IOPs 1, 2, 6 and 7. During these IOPs the median of *h*_jet_ determined from the fitted profiles (Equations 2 and 3) was between 15 and 25 m above ground.

The flow shows a number of similarities with shallow katabatic flows (e.g. Nadeau *et al*., 2013; Oldroyd *et al*., 2014; Grachev *et al*., 2016): a well‐developed low‐level jet, with strong shear below *h*_jet_; strongest TKE and negative momentum flux and slope‐normal heat flux near the surface decreasing in magnitude with height as they approach *h*_jet_. These similarities mostly relate to the near‐surface turbulence structure. The mean structure, however, does show some important differences. The jet maximum is embedded within the strong elevated inversion layer, as commonly observed for other deeper katabatic flows, for example, in Vertical Transport and MiXing (VTMX: e.g. Poulos *et al*., 2000; Monti *et al*., 2002; Heinemann, 2004; Zhong and Whiteman, 2008), while the stability in the near‐surface inversion is significantly weakened. The wind turning with height within the tower depth can be as much as 80°. At the same time, there is no consistent directional change with time, pointing to the fact that the observed phenomena are indeed katabatic flows and not nocturnal low‐level jets (cf. Shapiro *et al*., 2016). The shallower flows reported in literature (e.g. Oldroyd *et al*., 2014; Grachev *et al*., 2016) are mostly unidirectional and the jet maximum is found at the top of the near‐surface inversion, capped by weaker stability.

The largest difference to shallow flows, however, lies in the turbulence structure above *h*_jet_. Where shallow katabatic flows commonly display well‐developed turbulence (cf. Grisogono and Oerlemans, 2002; Nadeau *et al*., 2013; Grachev *et al*., 2016; Oldroyd *et al*., 2016), fluxes above *h*_jet_ are near zero in these deep katabatic flows. The exception is IOP6, where the momentum flux becomes positive above *h*_jet_, and TKE and the slope‐normal heat flux have a secondary maximum/minimum. However, tethered balloon profiles from this IOP show that a secondary jet with a wind speed maximum at around 140 m (not shown) was responsible for this significant, temporally variable mixing above the primary jet (seen also in the large shaded area in Figure 3), so that this IOP is not representative of characteristics of undisturbed katabatic flows and will not be studied in detail in the rest of the article.

**Figure 4:**
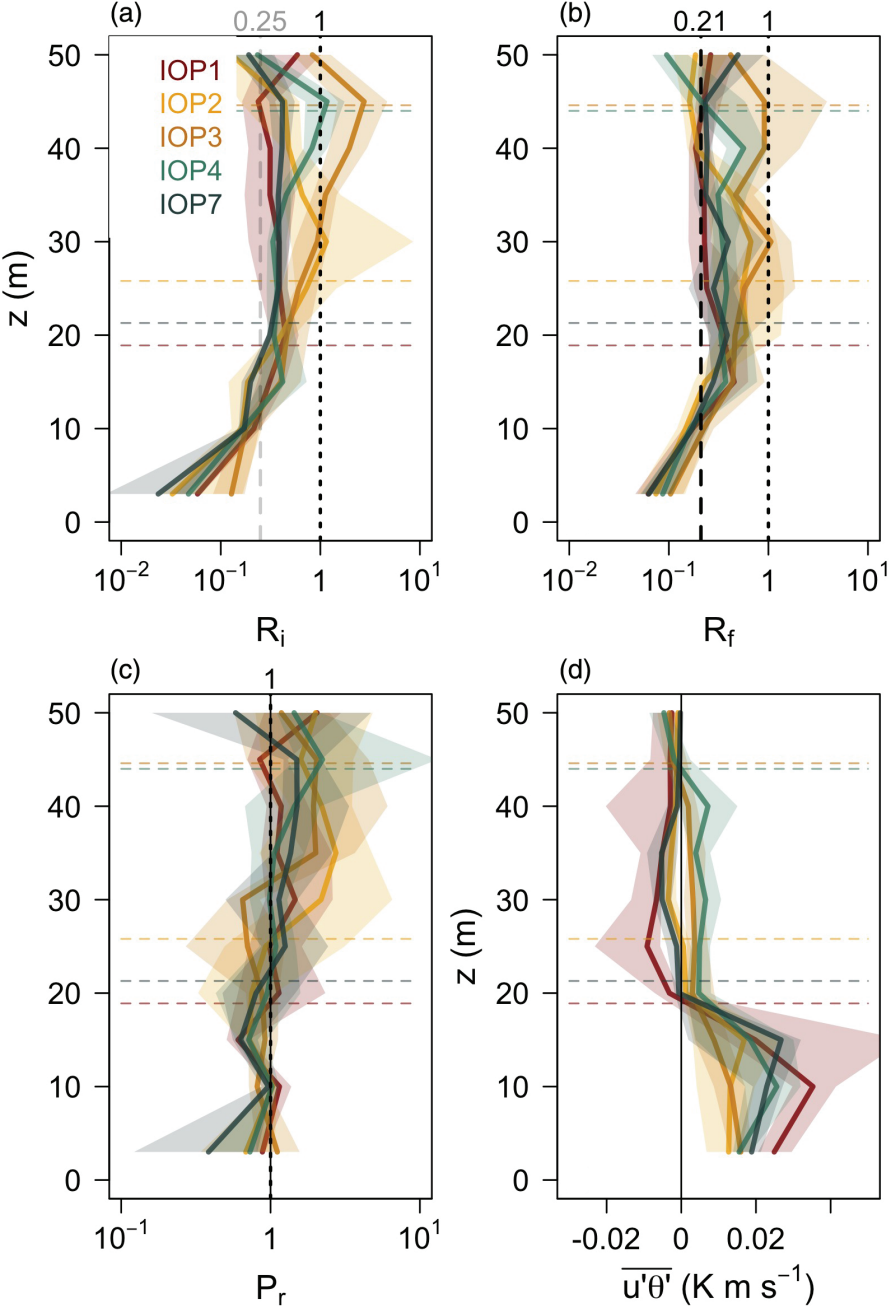
Median profiles of (a) gradient Richardson number (R_i_), (b) flux Richardson number (R_f_), (c) turbulent Prandtl number (P_r_), and (d) streamwise heat flux for IOPs 1, 2, 3, 4 and 7 calculated between 2200 and 0000 MST. Shading shows the interquartile ranges. Horizontal dashed lines indicate medians of the *h*_jet_ for each IOP [Colour figure can be viewed at wileyonlinelibrary.com]

While we can see that the height where fluxes go to zero coincides approximately with the mean jet height, the near‐zero fluxes above *h*_jet_ are related to the immersion of the jet maximum within the inversion (cf. Figure 3a). Figure 4 shows that profiles of gradient (*R*_*i*_) and flux (*R*_*f*_) Richardson numbers increase with height in a similar manner for all IOPs. Above the jet maximum height *R*_*f*_ increases beyond a critical value, commonly accepted to be between 0.21 and 0.25 (cf. Freire *et al*., 2019b), indicating in this simple one‐dimensional way that the buoyancy destruction of turbulence is larger than shear production at these heights. It is interesting to note that *R*_*i*_ shows strikingly similar behavior to *R*_*f*_, despite there being no accepted critical *R*_*i*_ (cf. Mauritsen *et al*., 2007; Canuto *et al*., 2008). This similarity is also visible in the profiles of the turbulent Prandtl number (Figure 4c) which are comparatively close to one, as also observed in Parmhed *et al*. (2004). Still, the height at which *R*_*f*_ exceeds 0.21 does not perfectly match *h*_jet_ (dashed lines in Figures 3 and 4) or the height where fluxes become insignificant (e.g. Figure 3c–e). Rather, it is somewhat lower, coinciding with the lower boundary of the elevated high‐stability layer and tops the region where fluxes have their largest value.

**Figure 5:**
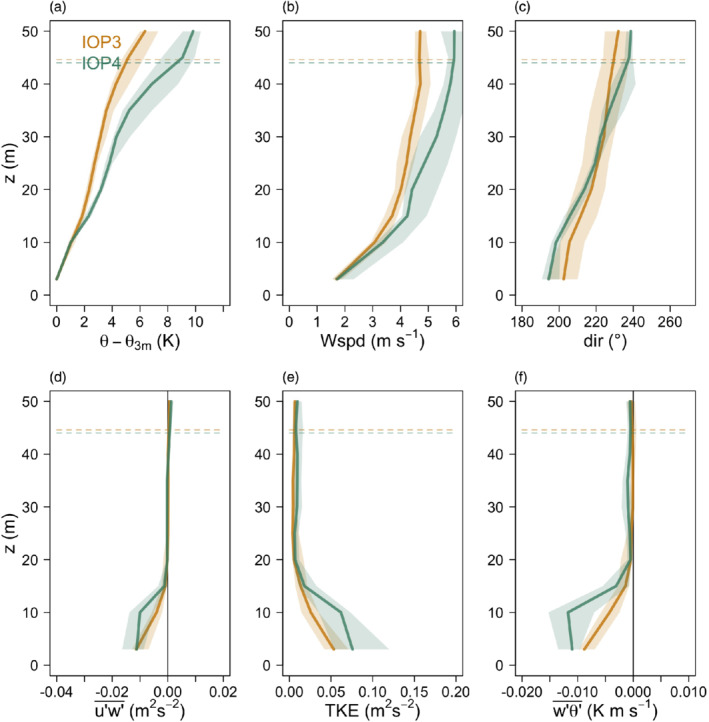
Same as Figure 2, but for IOPs 3 and 4 [Colour figure can be viewed at wileyonlinelibrary.com]

While *h*_jet_ for IOPs 1, 2, 6 and 7 was between 15–25 m (Figure 3), for IOPs 3 and 4 it was approximately 40 m (Figure 5). That these profiles do have a jet structure was additionally verified by tethered‐balloon soundings (not shown). Comparison between these two sets of IOPs shows the differences in their mean characteristics: the inversion in the first 35 m is significantly weaker in the deeper than in the shallower IOPs and therefore produces a less pronounced and wider jet maximum. Wind direction also changes less with height (40°) compared to the shallower IOPs. On the other hand, the turbulence structure is surprisingly similar. Despite the jet maximum being located at around 40 m, the streamwise momentum fluxes approach zero already at 15 m, the same as for the shallower IOPs. The TKE and slope‐normal heat flux, however, have a minimum at 20 m. As we already saw, the location where the fluxes start to become insignificant is slightly above the height where *R*_*f*_ exceeds 0.21 (Figure 4). It is clear from these results that *h*_jet_ is not the relevant length‐scale for the turbulence structure of the deeper IOPs. Normalizing the vertical scale by *h*_jet_ (as for example in Denby and Smeets, 2000) therefore does not collapse the turbulence profiles of the different IOPs. Which other relevant length‐scale is governing the katabatic dynamics will be explored further in Section 5.

The shape of the jet and the turbulence structure of the deeper katabatic‐flow IOPs (3 and 4) raise the question of whether these deeper flows represent a superposition of multiple katabatic flows (cf. Monti *et al*., 2002). The similarity in the turbulence structure of the deeper and shallower katabatic flows and the difference in the potential temperature profiles (weaker stability above 20 m for deeper flows) seem to suggest this. This hypothesis, however, cannot be validated and the origin of the superimposed upper‐level flow cannot be determined from the available data.

### 3.2 Scale‐wise analysis

Interestingly, unlike other fluxes, the streamwise heat flux ($\bar{u^{\prime }\theta^{\prime }}$) is non‐zero even above the height where other fluxes go to zero (Figure 4d). It does, however, change its sign at *h*_jet_, at around 20–25 m for shallower IOPs and 40 m for deeper ones, as also observed by Grachev *et al*. (2016). This change of sign at the jet maximum is commonly explained through the vertical profiles of temperature and wind speed, and therefore is a result of correlation between *u*, *w* and *θ* (e.g. Grachev *et al*., 2016; Tampieri, 2017). The fact that the streamwise heat flux is non‐zero above around 20 m, unlike all other fluxes, points to it being predominantly caused by other mechanisms of potentially non‐turbulent nature that still retain the same relation between *u*, *w* and *θ*. The non‐turbulent motions reflected in horizontal wind variability on scales between 1 and 30 min are commonly defined as sub‐mesoscale motions (cf. Vercauteren *et al*., 2016).

**Figure 6:**
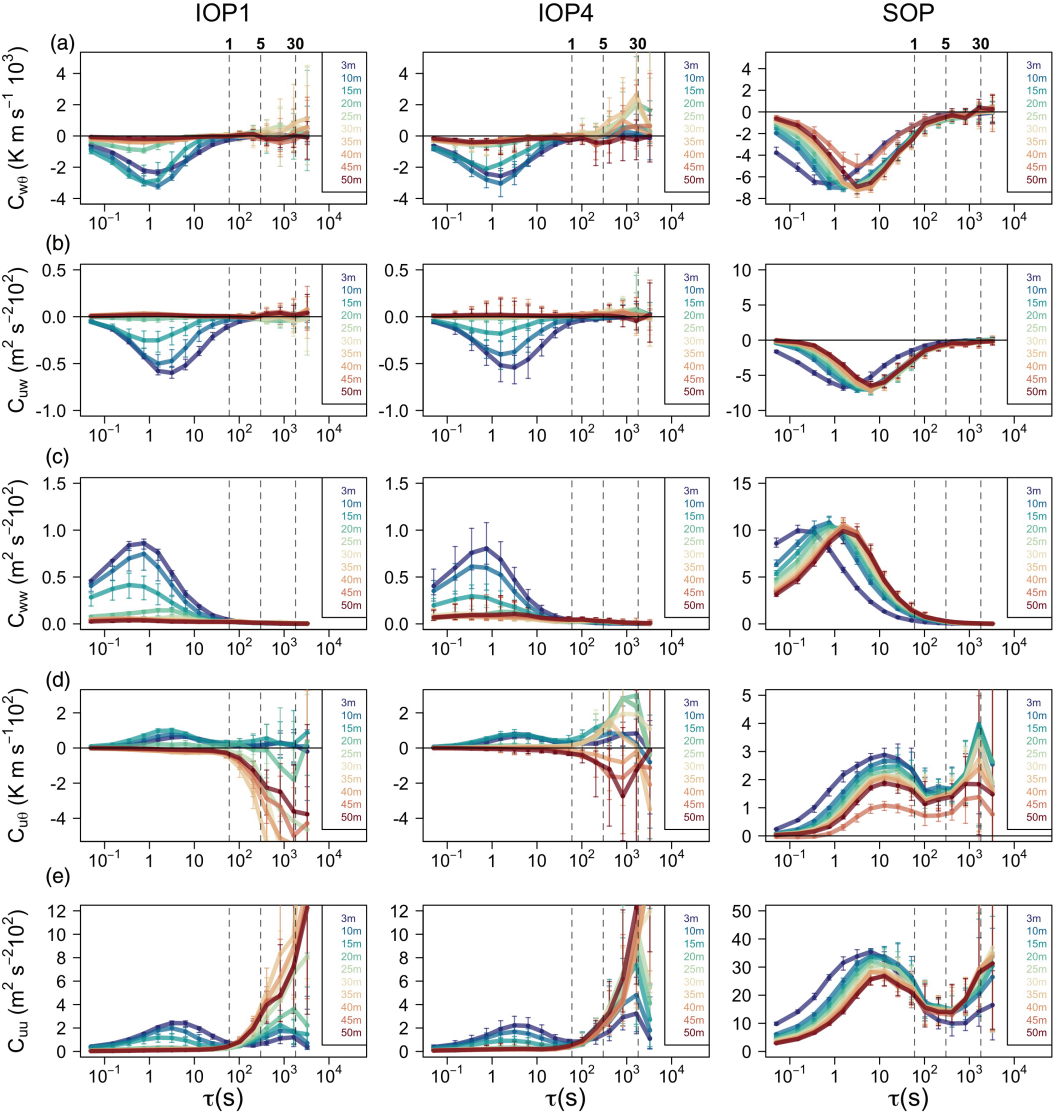
Multi‐resolution flux decomposition of (a) slope‐normal heat flux, (b) streamwise momentum flux, (c) slope‐normal velocity variance, (d) streamwise heat flux and (e) streamwise velocity variance for IOPs 1 and 4 and SOP for each measurement height. Thick lines correspond to medians and error bars to the interquartile range calculated over the entire night‐time period of the IOPs. Vertical dashed lines indicate the 1, 5 and 30 min time‐scales. Note the different *y*‐axis scales for the IOPs and the SOP [Colour figure can be viewed at wileyonlinelibrary.com]

We use the MRD analysis (Figure 6) to explore the contributions of these sub‐mesoscale motions to the total flux at each scale. While both the streamwise momentum flux, slope‐normal heat fluxes and slope‐normal velocity variance have maximum flux contributions at turbulent scales and near‐zero contributions at around one‐ to five‐minute scale, the sub‐mesoscale contributions to the streamwise heat flux are significant already at the one‐minute scale. Similarly, MRD shows that the streamwise velocity variance not only has a maximum at turbulent scales but also a secondary maximum at around 30 min, particularly above *h*_jet_. Interestingly, the secondary maximum at sub‐mesoscales is also present in the synoptically driven SOP; however, there the dominant peak in the co‐spectrum is still in the turbulence range and there appears to be a clear energy gap between the turbulence and non‐turbulent scales.

We can further examine the scale‐wise tendency of the fluxes with height. The sub‐mesoscale contribution to slope‐normal momentum flux, heat flux and velocity variance is almost negligible both below and above the jet maximum, therefore suggesting that these turbulence statistics are not influenced by sub‐mesoscale variability at higher levels. Similarly, Parmhed *et al*. (2004) found that katabatic flow is dynamically decoupled from the flow above, which they explain through the Scorer parameter. For streamwise velocity variance and streamwise heat flux, on the other hand, the sub‐mesoscale contribution is non‐negligible but close to the surface it is smaller than the turbulent contribution. As we approach the jet maximum height or the height where *R*_*f*_ = 0.21, however, this sub‐mesoscale contribution begins to dominate the turbulence statistics, suggesting significant and consistent horizontal wind variability.

As expected, the variability of sub‐mesoscale fluxes is significantly larger than that of turbulent fluxes. Surprisingly, however, the sign and tendency of the sub‐mesoscale flux is rather systematic with height (especially for IOP1) and is consistent with the expectation that the flux changes sign at the jet maximum. Identifying the origin of the non‐turbulent sub‐mesoscale motions superimposed on the katabatic flow is beyond the scope of this article.

### 3.3 Turbulence budgets

Next we investigate the momentum, TKE, and streamwise heat flux budgets in order to isolate the processes responsible for the observed flow structure. Due to the limitations of the measurement design, only the vertical gradient terms can be assessed confidently. We excluded the vertical advection terms from the budgets due to the large uncertainty in the estimates of mean slope‐normal velocity from the planar fit method. In the momentum budget, however, we can estimate the along‐slope pressure gradient from the near‐surface pressure measurements at the FAR and NEAR towers. The pressure measurements were first hydrostatically adjusted to the height of the NEAR tower by using the mean temperature between the two sites.

The examined budgets in sloped coordinates are described by the following equations. Following Haiden and Whiteman (2005), the momentum budget of the downslope component can be written as:

(9) $$\begin{array}{cc} 0 & =\underset{U_{I}}{\underbrace{-\frac{\partial U}{\partial t}}}\underset{U_{\mathrm{II}}}{\underbrace{-U\frac{\partial U}{\partial x}}}\underset{U_{\mathrm{III}}}{\underbrace{+g\frac{\Delta \theta }{\theta_{o}}\sin\alpha_{u}}}\underset{U_{\mathrm{IV}}}{\underbrace{-\frac{1}{\rho_{o}}\frac{\partial p}{\partial x}}}\\ & \;\underset{U_{V}}{\underbrace{-\frac{\partial \bar{u^{\prime }w^{\prime }}}{\partial z}}}\underset{U_{\mathrm{VI}}}{\underbrace{+\text{fV}\cos\alpha_{u}}}+R. \end{array}$$

The momentum budget equation for the across‐slope component is given by (expanded from Stiperski *et al*., 2007):

(10) $$0=\underset{V_{I}}{\underbrace{-\frac{\partial V}{\partial t}}}\underset{V_{\mathrm{II}}}{\underbrace{-U\frac{\partial V}{\partial x}}}\underset{V_{\mathrm{III}}}{\underbrace{+g\frac{\Delta \theta }{\theta_{o}}\sin\alpha_{v}}}\underset{V_{\mathrm{IV}}}{\underbrace{-\frac{\partial \bar{v^{\prime }w^{\prime }}}{\partial z}}}\underset{V_{V}}{\underbrace{-\text{fU}\cos\alpha_{v}}}+R.$$

The TKE budget equation is defined following Oldroyd *et al*. (2016):

(11) $$0=\underset{{\text{TKE}}_{I}}{\underbrace{-\frac{\partial \text{e}}{\partial t}}}+\frac{g}{\theta_{o}}\left(\underset{{\text{TKE}}_{\mathrm{II}}}{\underbrace{-\bar{u^{\prime }\theta^{\prime }}\sin\alpha_{u}}}\underset{{\text{TKE}}_{\mathrm{III}}}{\underbrace{-\bar{v^{\prime }\theta^{\prime }}\sin\alpha_{v}}}\underset{{\text{TKE}}_{\mathrm{IV}}}{+\underbrace{\bar{w^{\prime }\theta^{\prime }}\cos\alpha }}\right)$$

$$\underset{{\text{TKE}}_{V}}{\underbrace{-\bar{u^{\prime }w^{\prime }}\frac{\partial U}{\partial z}}}\underset{{\text{TKE}}_{\mathrm{VI}}}{\underbrace{-\bar{v^{\prime }w^{\prime }}\frac{\partial V}{\partial z}}}\underset{{\text{TKE}}_{\mathrm{VII}}}{\underbrace{-\bar{w^{\prime }w^{\prime }}\frac{\partial W}{\partial z}}}\underset{{\text{TKE}}_{\text{VIII}}}{\underbrace{-\frac{\partial \bar{w^{\prime }e^{\prime }}}{\partial z}}}\underset{{\text{TKE}}_{\mathrm{IX}}}{\underbrace{-\epsilon }}+R.$$

Finally, the streamwise heat flux budget is defined as:

(12) $$0=\underset{{\text{UT}}_{I}}{\underbrace{-\frac{\partial \bar{u^{\prime }\theta^{\prime }}}{\partial t}}}\underset{{\text{UT}}_{\mathrm{II}}}{\underbrace{+g\frac{\bar{\theta^{\prime 2}}}{\theta_{o}}\sin\alpha_{u}}}\underset{{\text{UT}}_{\mathrm{III}}}{\underbrace{-\bar{w^{\prime }\theta^{\prime }}\frac{\partial U}{\partial z}}}\underset{{\text{UT}}_{\mathrm{IV}}}{\underbrace{-\bar{u^{\prime }w^{\prime }}\frac{\partial \theta }{\partial z}}}\underset{{\text{UT}}_{V}}{\underbrace{-\frac{\partial \bar{w^{\prime }u^{\prime }\theta^{\prime }}}{\partial z}}}+R.$$

Here *R* is the residual of each budget,* e* is the TKE, *ρ*_*o*_ is the mean density in the layer below the measurement height, *g* is the acceleration due to gravity, *f* is the Coriolis parameter for the latitude 35°N, and angles *α*_*u*_ and *α*_*v*_ are defined by Equations 6 and 7. The temperature deficit Δ*θ* in the momentum budgets (*U*_III_and *V*_III_ in Equations 9 and 10) was calculated as the departure of the measured potential temperature profile from the one obtained by extrapolating the free tropospheric potential temperature gradient toward the surface. Along‐slope advection was only calculated for mean quantities due to a large separation distance between the towers, and it is therefore not present in the budgets of TKE (Equation 11) and streamwise heat flux (Equation 12).

**Figure 7:**
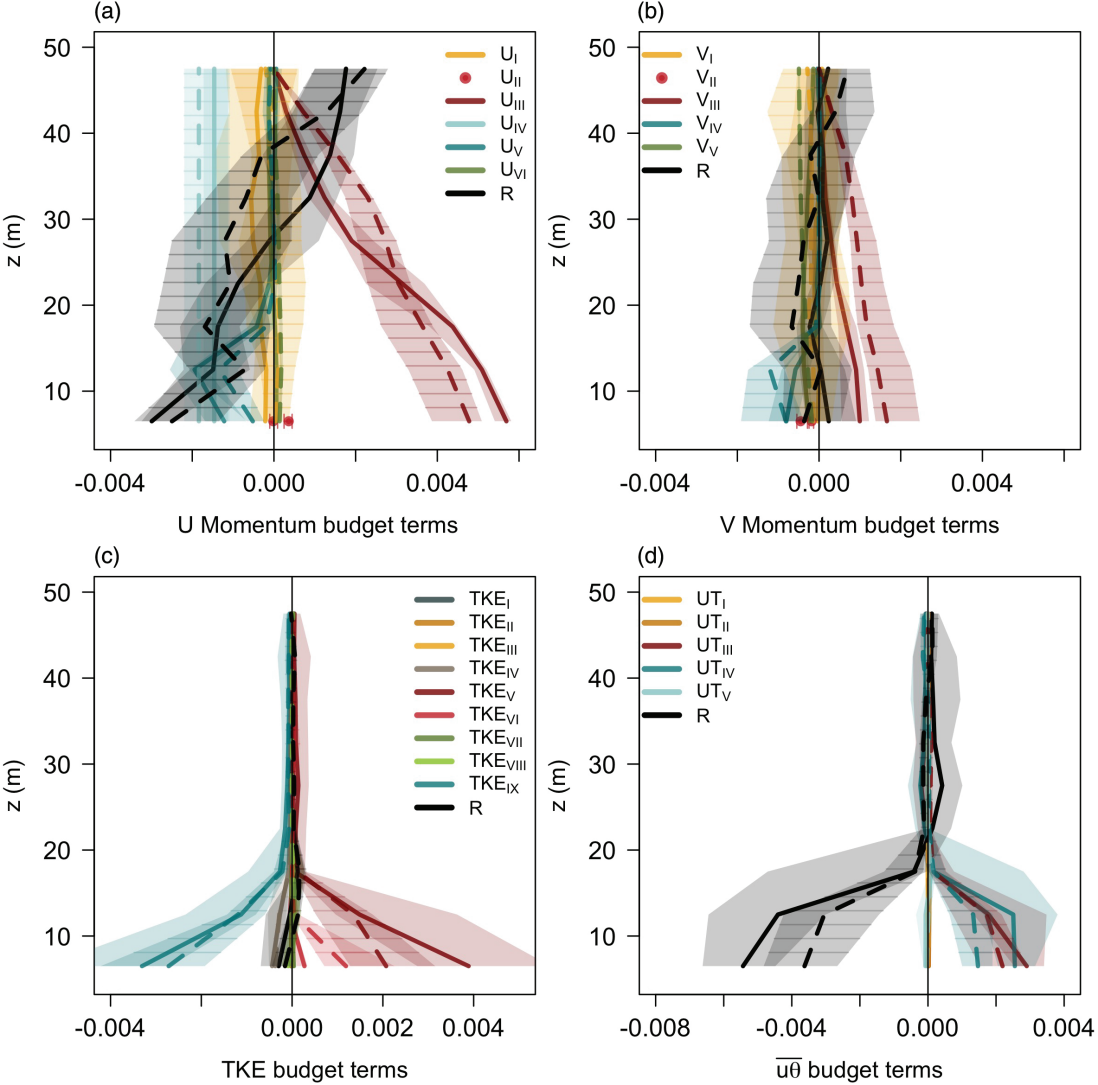
Median vertical profiles between 2200 and 0000 MST of (a) streamwise momentum, (b) spanwise momentum, (c) TKE and (d) downslope heat flux budgets terms for IOP1 (full line) and IOP4 (dashed line). Shading represents the interquartile range, and for IOP4 is additionally shown with horizontal stripes. The pressure gradient term in the momentum budget is estimated at the lowest level from the pressure difference between the NEAR and FAR towers and is assumed to be constant with height [Colour figure can be viewed at wileyonlinelibrary.com]

The budgets for a deeper (IOP4) and a shallower (IOP1) katabatic case are illustrated in Figure 7. The streamwise momentum budget (Figure 7a) shows that, as expected, the flow is forced by negative buoyancy (*U*_III_) oriented down the slope, caused by the strong near‐surface temperature deficit. Momentum transport toward the surface (*U*_V_) and the pressure gradient (*U*_IV_) both act to retard the flow near the surface leading to the jet structure, similarly to what was found by Poulos *et al*. (2000). The flow, however, is unsteady, with the non‐zero storage term (*U*_I_) increasing in magnitude with height for the shallower case. This corresponds to an increase of katabatic strength with time, but also oscillations in its strength. Such unsteadiness is also found in the solutions of the non‐linear Prandtl model (cf. Güttler *et al*., 2016). The largest difference in the budget between the deeper and shallower case is in the buoyancy forcing, which above 20 m is larger for the deeper case than for the shallower IOP1, which in its turn has a larger positive residual. The buoyancy forcing integrated over the tower depth on the other hand is of similar magnitude for both cases. Comparing our results to the classification of gravity flows as defined by Mahrt (1982) we can conclude that flow in both shallower and deeper IOPs is a shooting flow within the classification of stationary flows with negligible Coriolis effect.

Whilst the Coriolis force is irrelevant for the streamwise component (*U*_VI_), it is non‐negligible in the spanwise momentum budget (Figure 7b, *V*_V_) and its contribution becomes particularly relevant above approximately 20 m height (cf. Stiperski *et al*., 2007). Due to the orientation of the coordinate system and the change of wind direction with height, there is a significant contribution from buoyancy (*V*_III_) and from the momentum flux divergence (*V*_IV_) to the spanwise momentum budget that would not exist if the flow was perfectly aligned with the slope. While the along‐slope advection terms (*U*_II_, *V*_II_) for both budgets are negligible, it is impossible to assess the advection of the across‐slope component that could play a role in the momentum budget. Still, the momentum budgets particularly for the streamwise momentum component are not closed, suggesting important processes that are unaccounted for. Some of the source of uncertainty that could explain the under‐closure come from the estimate of the pressure gradient term, as well as the background stratification.

It is interesting to see the amount of similarity between the turbulent budgets of these two IOPs despite the large differences in their mean structure. The TKE budget (Figure 7c) confirms that near‐surface turbulence is shear driven. Here, contributions from both the streamwise (*TKE*_V_) and spanwise (*TKE*_VI_) momentum fluxes are important, especially for deep cases. Turbulence production is balanced by turbulent dissipation (*TKE*_IX_) (cf. Horst and Doran, 1988) and the negative slope‐normal heat flux (*TKE*_IV_) acting to suppress turbulence. The contribution from the streamwise heat flux to TKE generation/destruction (Oldroyd *et al*., 2016) is negligible due to the very low slope angle. Above about 20 m height, the TKE budget terms show negligible turbulence generation or destruction, but their ratio (as shown in *R*_*f*_) shows the dominance of buoyancy suppression (Figure 4). The contribution of horizontal shear generation to TKE (cf. Goger *et al*., 2018) could not be estimated from our dataset; however, given the extensiveness of the slope we can speculate that its contribution is not dominant.

The budget of the streamwise heat flux (Figure 7d) shows similarity with the TKE budget, in terms of negligible contributions of the budget terms above 20 m for both IOPs, despite the streamwise heat fluxes being non‐negligible at these heights (cf. Figure 4). This points to non‐local sources of streamwise heat fluxes. Below 20 m the dominant positive contributions come from the vertical wind shear (*UT*_III_) and vertical temperature gradient (*UT*_IV_) terms. Interestingly, while the shear term (*UT*_III_) is similar for both IOPs, the temperature gradient term (*UT*_IV_) is significantly smaller for the deeper case. This is due to the deeper case having weaker near‐surface stability. The turbulence suppression due to buoyancy does not play a significant role, contrary to results for steeper slopes. The budget is also significantly under‐closed.

## 4 STABLE BOUNDARY‐LAYER HEIGHT

The availability of turbulence measurements with a high vertical resolution reaching beyond the jet‐maximum allows us to detect the SBL top directly from turbulence measurements without having to resort to indirect estimates such as using the vertical temperature and wind speed gradients (cf. Heinemann, 2004; Fedorovich and Shapiro, 2009). Under the assumption that turbulence ceases above the BL, we can define the SBL height as that height where small‐scale turbulent fluxes first become insignificant. Note that, due to the fact that our method for determining SBL height starts from the surface upwards, our methodology will potentially only detect the turbulent BL below *h*_jet_. In reality, and particularly for shallow katabatic flows, turbulence can develop again above the katabatic jet maximum. Whether this turbulence can still be considered as BL turbulence (see the definition of the boundary layer in Stull (1988)) though remains an open question. The scaling results of Grachev *et al*. (2016) conforming with *z*‐less scaling, however, confirm that above the jet maximum turbulence is decoupled from the surface.

Even though the vertical resolution of the measurements is unprecedented, finding the exact SBL height is subject to some ambiguity and we therefore use an ensemble approach. First, profiles of streamwise momentum flux, slope‐normal heat flux and TKE were linearly interpolated between the measurement levels and then the height (*z*) where the flux profiles fell below a minimum value was identified:

$$h_{\text{uw}}=z\left(\bar{u^{\prime }w^{\prime }}>-0.001\;m^{2}\cdot s^{-2}\right),$$

$$h_{\text{w}\theta }=z\left(\bar{w^{\prime }\theta^{\prime }}>-0.001\;K\cdot m\cdot s^{-1}\right),$$

$$h_{\text{TKE}}=z\left(\text{TKE}<0.1\;m^{2}\cdot s^{-2}\right).$$

We have also tested an alternative SBL height estimate using the height at which the fluxes fall below 10 or 1% of their surface (3 m) value (cf. Caughey *et al*., 1979). The heights determined that way, however, deviated from each other more significantly than if hard limits were used (e.g. the correlation coefficient between *h*_*TKE*_ and *h*_*wθ*_ is 0.84 when using hard limits, and 0.58 when using percentage of surface value). We finally calculated the median of these heights and use it as the ensemble SBL height:

(13) $$h_{\mathrm{SBL}}=\text{median}\left(h_{\text{uw}},h_{\text{w}\theta },h_{\text{TKE}}\right).$$

To further reduce the uncertainty in the SBL height estimation we smoothed the data using a six‐point moving average.

**Figure 8:**
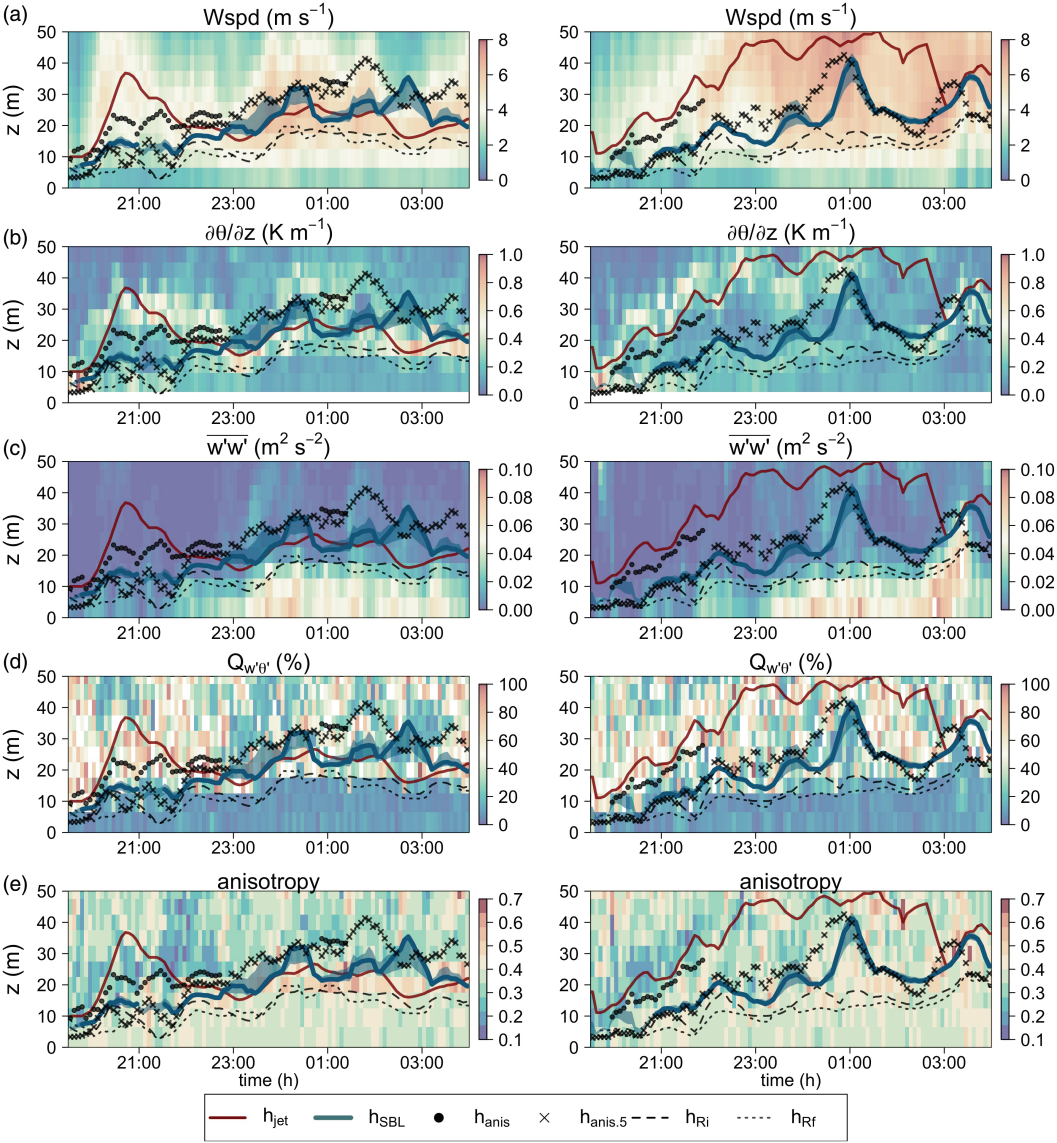
Time–height diagrams of (a) wind speed, (b) potential temperature gradient, (c) slope‐normal velocity variance, (d) stationarity of the heat flux, and (e) anisotropy invariant *y*_B_, for IOP1 (left) and IOP4 (right). The lines represent the heights of the maximum wind speed (*h*_jet_), the ensemble SBL height (*h*_SBL_) in thick line with the corresponding interquartile range in shading, the anisotropy height determined from one‐minute data (*h*_anis_) and five‐minute data (*h*_anis.5_), and heights where gradient (*h*_*Ri*_) Richardson number exceeds 0.25 and flux (*h*_*Rf*_) Richardson number exceeds 0.21. Time is in MST [Colour figure can be viewed at wileyonlinelibrary.com]

We first explore how the evolution of *h*_jet_, the ensemble SBL height (*h*_SBL_) and heights where *R*_*f*_ exceeds 0.21 (*h*_*Rf*_) and *R*_*i*_ exceeds 0.25 (*h*_*Ri*_) corresponds to the time–height evolution of downslope wind speed, potential temperature gradient, slope‐normal velocity variance (as a measure of turbulence intensity), stationarity of the heat flux and turbulence anisotropy (Figure 8). The spread around the median *h*_SBL_ corresponds to the interquartile range (25–75%) of the smoothed ensemble. The small spread of the ensemble shows that the height where fluxes become insignificant (Figure 8b) is consistent in time. This height is comparatively close to *h*_jet_ during the periods of well‐developed katabatic flow in IOP1 (2200–0300 MST). During IOP4 the height where fluxes become insignificant follows the time‐evolution of *h*_jet_ but it is significantly lower, as already seen in Figure 3. The jet maximum height closely corresponds to the height of the maximum temperature gradient (Figure 8b), but shows no systematic relation to the stationarity of turbulence (Figure 8d) or its anisotropy (Figure 8e). On the other hand, the SBL height shows no systematic relation to the temperature profile.

The fact that *h*_SBL_ does not correspond to either the jet height or the height of the inversion highlights the problems with using just the profiles of mean variables when defining SBL top for katabatic flows, as is commonly done (e.g. Van der Avoird and Duynkerke, 1999; Heinemann, 2004; Fedorovich and Shapiro, 2009). Despite the fact that there is no accepted critical *R*_*i*_, the two heights obtained from the gradient and flux Richardson numbers (*h*_*Ri*_ and *h*_*Rf*_) are surprisingly consistent, but are even lower than the SBL height. Interestingly, Richardson number heights appear to separate the regions with stationary turbulence (Figure 8d) more accurately than *h*_SBL_. Similarly, together with SBL height, both Richardson number heights seem to separate close‐to‐isotropic near‐surface turbulence (Figure 8e) from the highly non‐stationary more anisotropic turbulence above. This is intuitive since turbulence becomes more anisotropic in very stable conditions where the influence of sub‐mesoscale motions is also significant (cf. Stiperski and Calaf, 2018; Vercauteren *et al*., 2019) or above the BL where geostrophic turbulence is two‐dimensional. In the initiation phase of the katabatic flow, these very stable conditions with anisotropic turbulence are found also very close to the ground as already observed by Banta ( 2008). This prompts us to introduce an alternative SBL height diagnostic based on anisotropy.

We define the anisotropy height *h*_anis_ (filled circles in Figure 8) as that height where the vertical coordinate of the barycentric Lumley triangle (*y*_B_) falls below a critical value. After several tests (not shown) we determined that $y_{\mathrm{Bc}}=\sqrt{3}/6$, for which the velocity aspect ratio falls below 0.3 (Mahrt *et al*., 2012), is the most appropriate critical value for separating quasi‐isotropic BL turbulence and the anisotropic turbulence above the SBL. Using just *y*_B_ to define anisotropy is in accordance with the results of Brugger *et al*. (2018) and Stiperski *et al*. (2019) who showed that distance to isotropy is the most relevant anisotropy invariant for atmospheric turbulence.

Since the criterion for determining the anisotropy height was not always met using the one‐minute averages (there are periods when anisotropy does not fall below the chosen critical value within the tower depth), we additionally tested an alternative anisotropy height determined from the fluxes in which turbulent deviations were defined at a 5 min scale. As mentioned previously, BL turbulence is still well‐developed at the 5 min scale and therefore the 5 min anisotropy will still reflect turbulence topology. On the other hand, above the SBL the sub‐mesoscale contribution is already significant at the 1 min scale, therefore the 5 min anisotropy will reflect the topology of sub‐meso motions. This alternative anisotropy height (*h*_anis.5_) is indicated by crosses in Figure 8. We can see that both anisotropy heights suggest that the SBL might be deeper than indicated by *h*_SBL_ although the three measures do coincide in certain periods both for the shallower and deeper katabatic cases. The discrepancy between the different measures used to detect the actual SBL height indicates that the actual SBL height itself might not always be uniquely defined.

Given the low slope angle and weak relation between the SBL height and *h*_jet_, we are interested to know if this katabatic SBL resembles the one over flat terrain and if the flat‐terrain formulations of SBL height can correctly predict the katabatic SBL height. We therefore examine the correlation between the diagnosed SBL heights *h*_SBL_, *h*_anis_ and *h*_anis.5_ and several length‐scales defined in Table 1: equilibrium SBL heights *H*_EZ1_ according to Zilitinkevich and Baklanov (2002), *H*_EZ2_ according to Zilitinkevich *et al*. (2012), *H*_EM_ according to Mironov and Fedorovich (2010) and *H*_ES_ according to Steeneveld *et al*. (2007). These measures are flat‐terrain references that prioritize different processes such as background stratification, Coriolis effects or near‐surface heat flux in determining the SBL height. In addition, we test the buoyancy *H*_B_ and shear *H*_S_ length‐scales following Monti *et al*. (2002) based on the VTMX results for katabatic flows.

**Table 1:** List of height‐scales for the stable boundary layer

Variable	Name	Definition	Reference
*H*_EZ1_	Flat‐terrain equilibrium SBL height	$H_{\mathrm{EZ}1}=\frac{0.4u_{*s}}{\left|f\right|}{\left[1+\frac{0.4^{2}u_{*s}\left(1+0.25\text{NL}/u_{*s}\right)}{0.75^{2}\left|f\right|L}\right]}^{-1/2}$	Zilitinkevich and Baklanov (2002)
*H*_EZ2_	Flat‐terrain equilibrium SBL height	$H_{\mathrm{EZ}2}=0.5u_{*s}^{2}\cdot {\left|fB_{s}\right|}^{-1/2}$	Zilitinkevich *et al*. (2012)
*H*_ES_	Flat‐terrain equilibrium SBL height	$H_{\mathrm{ES}}=\left\{\begin{array}{c} 10u_{*s}\cdot N^{-1}\;u_{*s}^{2}N{\left|B_{s}\right|}^{-1}>10\\ 32{\left(\left|B_{s}\right|\cdot N^{-3}\right)}^{1/2}\;u_{*s}^{2}N{\left|B_{s}\right|}^{-1}<10 \end{array}\right.$	Steeneveld *et al*. (2007)
*H*_EM_	Flat‐terrain equilibrium SBL height	*H*_EM_ = u_*s_ · *N*^−1^(|*f*| · *N*^−1^)^−*1/2*^	Mironov and Fedorovich (2010)
*H*_B_	Buoyancy length‐scale	*H*_B_ = (*σ*_*w*_)_*s*_ · *N*^−1^	Monti *et al*. (2002)
*H*_S_	Shear length‐scale	HS=σws·∂U‾∂zs−1	Monti *et al*. (2002)
*H*_KAT_	Prandtl scale	*H*_KAT_ = 1/4 · u_*s_ · (*N*sin*α*)^−1^	
*H*_MRD_	MRD scale	$H_{\mathrm{MRD}}=2\pi \;\text{U}\tau_{max}$	

*Note*: *N* is the buoyancy frequency, u_*_ is friction velocity, *L* is the Obukhov length, *σ*_*w*_ is the standard deviation of vertical velocity, *f* the Coriolis parameter, *k* the von Kármán constant taken as 0.4, and *B*_*s*_ is buoyancy defined as $B_{s}={\left(\bar{w^{\prime }\theta^{\prime }}\right)}_{s}g/{\bar{\theta }}_{v}$, where ${\left(\bar{w^{\prime }\theta^{\prime }}\right)}_{s}$ is the kinematic sensible‐heat flux. Subscript *s* denotes values measured at the surface (i.e. 3 m).

An additional estimate of the depth of the katabatic flow can be obtained analytically using the linear formulation of the Prandtl model (cf. Shapiro and Fedorovich, 2014). We employ K‐theory and assume the eddy diffusivities for momentum (*K*_*m*_) and heat (*K*_*h*_) are equal and constant with height. This assumption is valid on average as the profile of the turbulent Prandtl number is close to one and does not show a consistent tendency with height (cf. Figure 4d). We can finally relate the eddy diffusivities to the friction velocity and the depth of the flow *K*_*h*_ = *K*_*m*_ *∼* u_*_ *H*_KAT_ to obtain:

(14) $$H_{\mathrm{KAT}}=C_{k}u_{*s}\cdot {\left(N\sin\alpha \right)}^{-1},$$

where *C*_*k*_ = 1/4 is a coefficient based on the Prandtl vertical scale (cf. Grisogono and Oerlemans, 2002). Finally, we define the MRD length‐scale *H*_MRD_ as the length‐scale of the eddy with the maximum contribution to the sensible‐heat flux (i.e. the length‐scale where ${\text{MRD}}_{w^{\prime }\theta^{\prime }}$ has a maximum). Since the MRD is performed in the time domain, we use Taylor's hypothesis to estimate this length‐scale from the time‐scale *τ*_max_ which corresponds to the time‐scale of the dominant eddy in ${\text{MRD}}_{w^{\prime }\theta^{\prime }}$. The MRD length‐scale is thus defined as:

(15) $$H_{\mathrm{MRD}}∼2\pi \;U\tau_{\max}.$$

**Figure 9:**
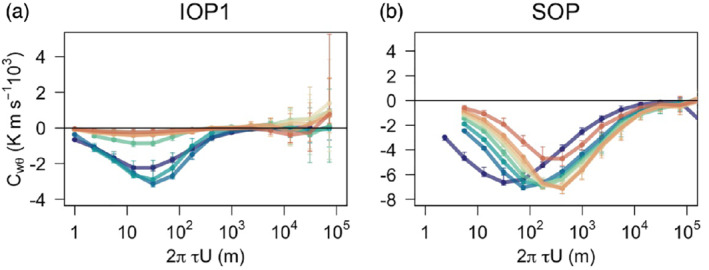
Multi‐resolution flux decomposition of heat flux co‐spectrum as a function of length‐scale for (a) IOP1, and (b) SOP. Colours represent heights and are the same as in Figure 6. Reference source not found [Colour figure can be viewed at wileyonlinelibrary.com]

Here *U* is the total wind speed. This length‐scale rests on the fact that for katabatic flows the MRD length‐scale of the dominant eddy is constant with height (Figure 9a). This constancy, consistent over different flux co‐spectra, suggests that turbulence in katabatic flow is not height‐dependent in a sense that the eddies contributing dominantly to the flux have a uniform size over the entire SBL. The same is not true for the MRD of the non‐katabatic SOP (Figure 9b) where the length‐scale of the dominant eddy is clearly height‐dependent, indicating that the distance from the surface is a relevant scaling variable, in line with surface‐layer scaling. This result already confirms that the turbulence structure of katabatic flows is inherently different from that of dynamically driven flows.

For a given dataset, having constant Coriolis parameter and slope angle, some of these length‐scales are obviously correlated since they are constructed from the same variables or variables that are themselves highly correlated (e.g. friction velocity and standard deviation of slope‐normal velocity). Examples are *H*_ES_ for weakly stable BL ($u_{s}^{*}N{\left|B_{s}\right|}^{-1}>10)$, the buoyancy scale *H*_B_ and the Prandtl katabatic depth *H*_KAT_. This fact justifies using the Prandtl katabatic depth despite the fact that the katabatic flows studied here do not conform to the classic linear Prandtl model.

Per definition, the buoyancy frequency used in Table 1 is the background free‐flow stability commonly estimated above the SBL or far above the katabatic flow. For the katabatic flows examined here, however, it is not entirely clear what this appropriate background stability should be (cf. Grisogono *et al*., 2015), given the layered nature of the potential temperature profiles (cf. Figures 3 and 5). We therefore compare the diagnostic SBL heights (*h*_SBL_, *h*_anis_ and *h*_anis.5_) to height‐scales calculated using *N* from three distinct layers: free‐flow background stratification of the larger‐scale environment (*N*_free_) calculated as an average *N* between 100 and 220 m obtained from the tethered‐balloon soundings, the maximum *N* within the inversion (*N*_max_), and the average *N* in the lowest 15 m (*N*_low_), the last two calculated from the tower measurements. To ensure an unbiased treatment of the data, we removed periods when the detected SBL height equalled the lowest measurement level (3 m) since we cannot ensure that the true SBL height is not below that level.

**Figure 10:**
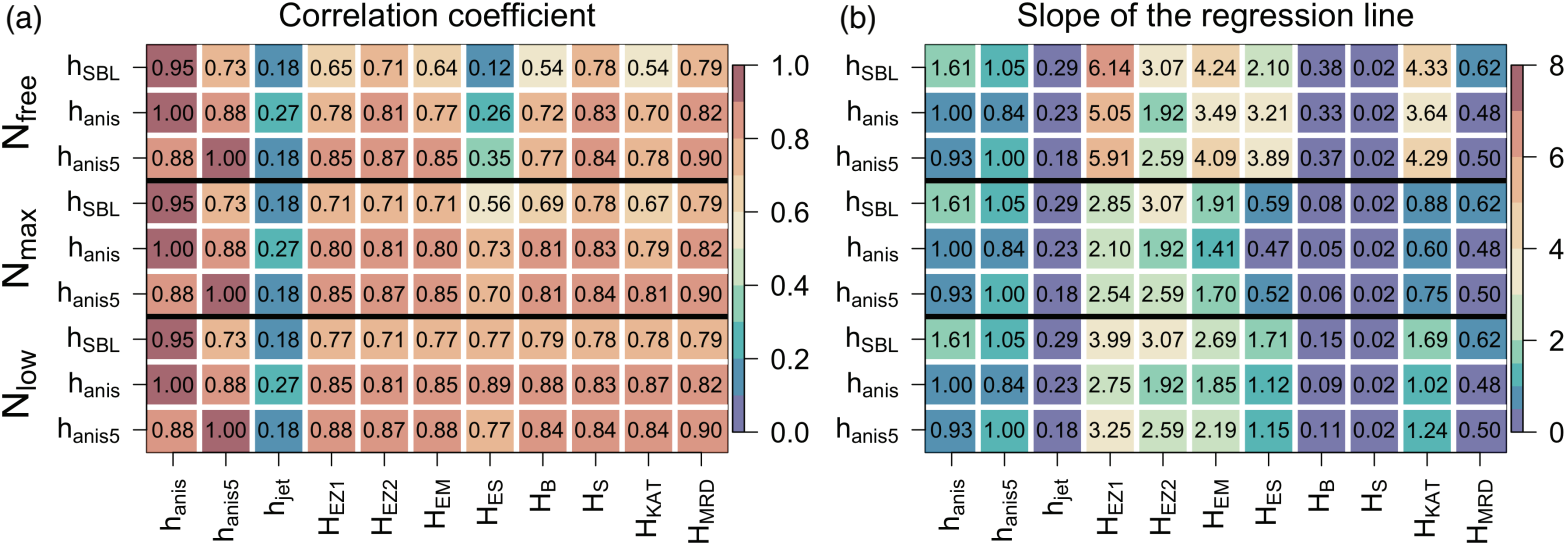
(a) Linear correlation coefficients, and (b) slope of the regression line calculated between the SBL (*h*_SBL_) and anisotropy heights (*h*_anis_, *h_anis.5_*) and different estimates of length‐scales defined in Table 1 for buoyancy frequency equal to *N*_free_, *N*_max_ and *N*_low_. Data are from the IOPs 1, 2, 3, 4 and 7, between 1930 and 0400 MST. Note that since *h*_jet_, *H*_EZ2_, *H*_S_ and *H*_MRD_ are independent of *N*, their values are the same for each set of rows [Colour figure can be viewed at wileyonlinelibrary.com]

The results of the correlation analysis (Figure 10a) show that two of the primary diagnostic heights, the anisotropy height (*h*_anis_) and the ensemble SBL height, are highly correlated (0.95), but the anisotropy height on a 5 min scale does not correlate particularly well with the SBL height (0.73) suggesting that it is an alternative independent estimate. The same is true for flat‐terrain estimates calculated using free tropospheric stability *N*_free_. They show quite poor correlation to both *h*_anis_ and *h*_SBL_. This suggests that the free tropospheric stability is not relevant in determining the depth to which the near‐surface turbulence can extend. On the other hand, the correlations increase considerably if the local stability is used instead, with the largest correlation for *N*_low_. Although this contradicts the results of flat‐terrain studies, it is intuitive since the jet maximum is mostly immersed within the inversion and therefore the flow has to work against that local stratification and not the free tropospheric stratification. The largest improvement by using local *N* is found for *H*_ES_ (correlation with *h*_SBL_ increasing from 0.11 to 0.78), although all of the data points are classified as strongly stable ($u_{*s}^{2}N{\left|B_{s}\right|}^{-1}<10$) irrespective of the chosen *N*. Generally, both of the anisotropy heights *h*_anis_ and *h*_anis.5_ correlate better than *h*_SBL_ with all of the examined length‐scales. There is also only limited variation in correlation coefficients between the different length‐scales, thus making it unclear which of the formulations is preferable.

Apart from correlation, however, we also want to see how well the formulations capture the estimated SBL height and therefore we examine the slope of the linear regression (Figure 10b), which indicates if the length‐scale is overestimating or underestimating the detected height. Interestingly, irrespective of the method used to diagnose SBL height and the way of determining buoyancy frequency (*N*_free,_ *N*_max,_ *N*_low_), all flat‐terrain equilibrium heights significantly overestimate the depth of the SBL by more than three and sometimes as much as six times (e.g. slope for *H*_EZ1_ calculated with *N*_free_ is 6.14). This suggests that, given the same forcing, a sloping SBL is significantly shallower than that over flat terrain. The exception here is *H*_ES_ which slightly underestimates the SBL height if using *N*_max_, but when using *N*_low_ and *h*_anis_ as a measure of SBL height it has an almost perfect regression slope of 1.12. Similarly, the katabatic length‐scale *H*_KAT_ has both a high correlation coefficient and appropriate slope of 1.02 making it the best candidate for the suitable analytical formulation of the katabatic SBL height. The buoyancy and particularly the shear scale, although well correlated to the diagnostic SBL heights, significantly underestimate them, which comes as no surprise given that they only represent a length‐scale. Interestingly, MRD scale (*H*_MRD_) with a high correlation coefficient (0.89) corresponds to close to half of the anisotropy heights (slope is 0.48). Although this length‐scale is not an analytical estimate of SBL, it appears to offer an easy way of determining SBL height from single‐level near‐surface turbulence fluxes in the absence of additional measurements at greater heights. The universality of this result, however, needs to be assessed with additional datasets.

**Figure 11:**
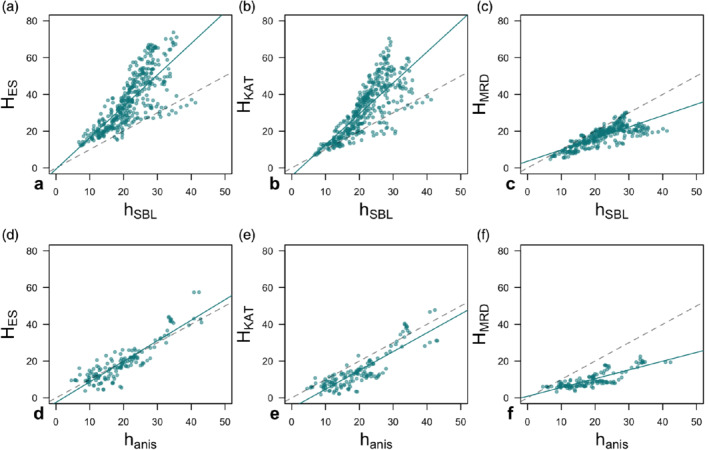
(a–f) Scatterplots of length‐scale estimates with the largest correlation coefficient (*H*_ES_, *H*_KAT_ and *H*_MRD_) calculated using *N*_low_ as function of SBL (*h*_SBL_) and anisotropy height (*h*_anis_). Data are from the IOPs 1, 2, 3, 4 and 7, between 1930 and 0400 MST. Dashed line is the perfect correlation curve, and the full line the linear regression line [Colour figure can be viewed at wileyonlinelibrary.com]

The results in Figure 10 allow us to cautiously draw two conclusions: one is on the more appropriate diagnostic height for katabatic SBL and the other is identifying the analytical SBL formulations that match that height. First, we can see that anisotropy heights appear to be the more appropriate measure for estimating the katabatic SBL height than *h*_SBL_, as the results show that the anisotropy heights are more correlated with analytical formulations of SBL height and additionally have a more uniform distribution of the residuals according to the Shapiro–Wilks test for the normality of the distribution of residuals (Wilks, 2011). This is clear also from scatterplots of *h*_SBL_, *h*_anis_ and the two top candidates for the best SBL estimate (*H*_ES_, *H*_KAT_) (Figure 11). Data are equally distributed around the best fit curve for *h*_anis_ but when using *h*_SBL_ two separate regimes with different regression slopes seem to exist. This suggests a mismatch in identifying all the relevant processes governing the flux‐driven SBL height. The major drawback in using *h*_anis_ on the other hand is that it is not always detectable at the 1 min scale; however, *h*_ani.5_ can be used instead. Second, of the analytical SBL heights (Table 1), several (*H*_ES_, *H*_B_, *H*_KAT_ and *H*_MRD_) have large correlation coefficients with *h*_anis_, but also have low RMSEs (4.2–5.2 m), low medians of the residuals (0.07–0.47 m), small offsets in the linear regression (−5 to −2.3 m), and residuals that are normally distributed. These additional diagnostics were computed from linear regression adjusted for the slope of the regression line from Figure 10b. Ultimately, without additional datasets from slopes of different steepness (varying *α*), latitudes (varying *f*) and SBL heights it is impossible to unequivocally establish which of these estimates (*H*_ES_, *H*_B_ or *H*_KAT_) is the most universal, how the constants in standard formulations of Table 1 need to be adjusted for the sloping SBL or indeed if the Coriolis force can be neglected. The results, however, do show that a successful predictor of SBL height will include information on local stability, a measure of the intensity of surface turbulence ($u_{s}^{*}$ or (*σ*_*w*_)_*s*_), and, likely, the slope angle *α*.

## 5 SCALING OF DEEP KATABATIC FLOWS

The fact that the SBL height can be directly detected from turbulence measurements provides an ideal opportunity to explore the different turbulence scaling regimes (Holtslag and Nieuwstadt, 1986) for these deep katabatic flows. Thus far no study has identified the existence of a surface layer in katabatic flows. Instead studies have shown that the fluxes vary considerably with height (e.g. Forrer and Rotach, 1997; Denby and Smeets, 2000; Grisogono and Oerlemans, 2001; Nadeau *et al*., 2013; Grachev *et al*., 2016). The reasons for this could be that most studies focused on shallow katabatic flows in which the surface layer would be too shallow to detect.

**Figure 12:**
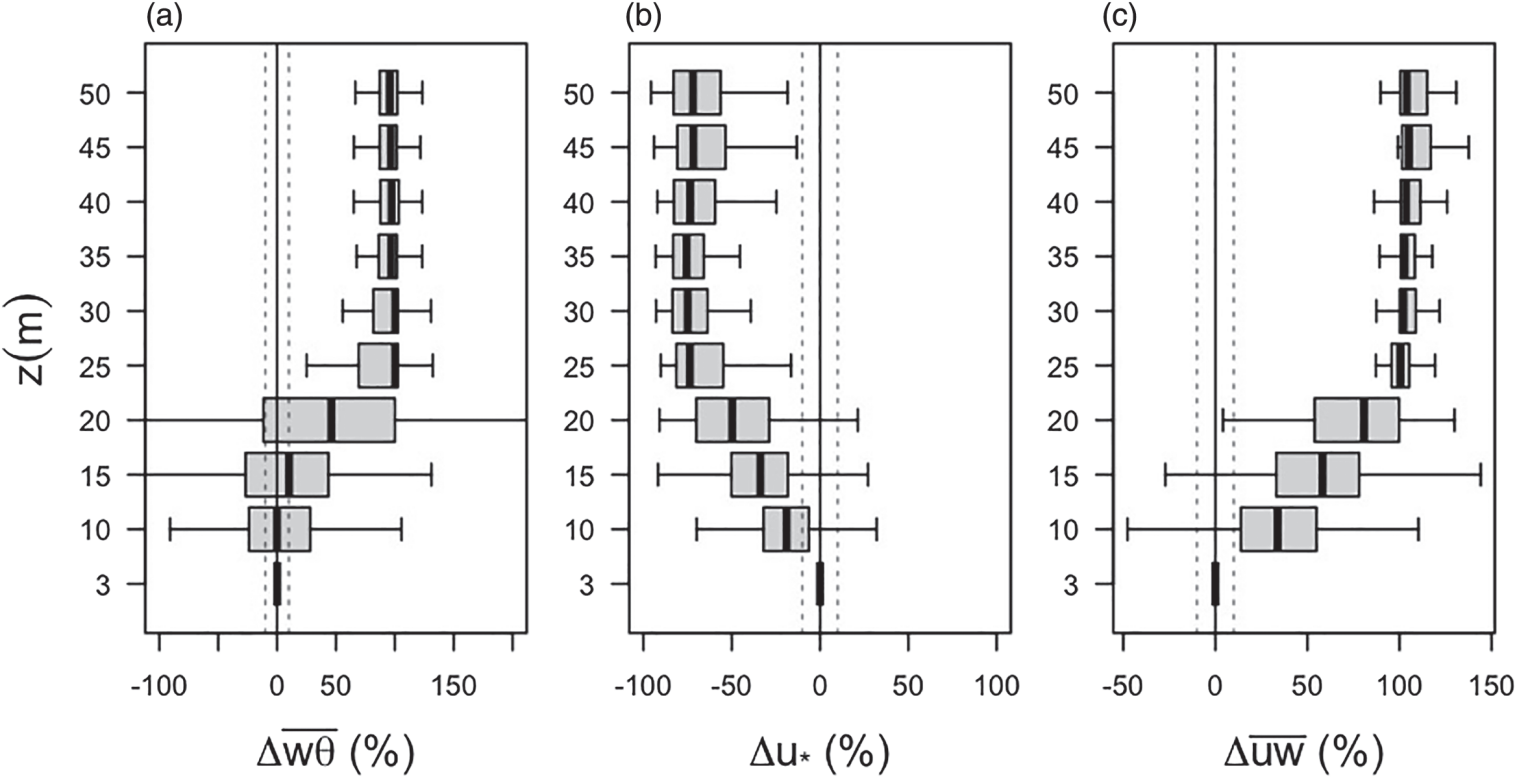
Normalized deviations of (a) slope‐normal heat flux, (b) friction velocity and (c) streamwise momentum flux as a function of height. The deviations are calculated as Δx = [(x(z) − x(z=3 m)]/|x(z=3 m)|*100, where *x*(*z*) is the value at height *z*, and is the value at the lowest measurement level. Shown are stationary data from all IOPs except IOP5. Vertical dashed lines indicate the 10% deviation from the surface flux value, corresponding to the commonly accepted amount of flux variation within the surface layer (cf. Stull, 1988)

Our results (Figure 12) show that even for the deepest flows studied here the momentum flux is non‐constant with height; however, the sensible‐heat flux is close to constant within the lowest two measurement levels (variations within 10% of the surface value). The lack of full flux constancy comes as no surprise since having constant fluxes up to 10 m height would mean that the SBL depth would be on the order of 100 m. Given that the median diagnosed SBL height over the periods with katabatic flow equals 20 m, but the individual maximum SBL height can be considerably higher at around 43 m, we could expect that at least at times the lowest measurement level at 3 m could be within the surface layer.

**Figure 13:**
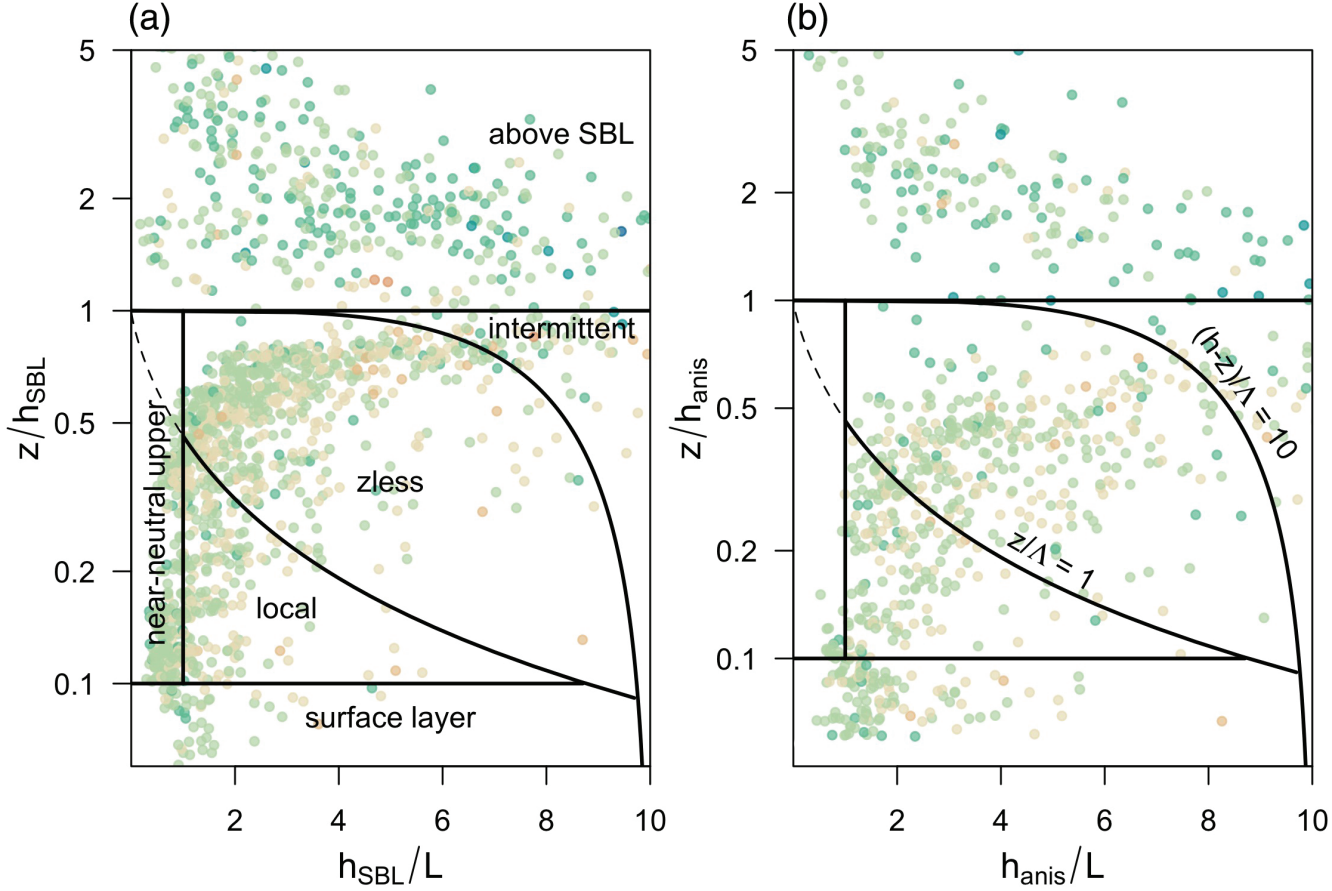
SBL scaling regimes according to Holtslag and Nieuwstadt (1986, their Fig. 2), for SBL height defined using (a) *h*_SBL_, and (b) *h*_anis_. Shown are stationary data from all IOPs except IOP5. Data points are coloured according to *y*_B_ (see colourbar in Figure 8e). Here *L* is the surface Obukhov length, whilst Λ is the local Obukhov length valid in the local and *z*‐less scaling regimes [Colour figure can be viewed at wileyonlinelibrary.com]

The distribution of the stationary data within the scaling diagram of Holtslag and Nieuwstadt (1986) confirms that some data points fall within the surface layer (Figure 13), when using both the *h*_SBL_ and *h*_anis_ as the diagnostic SBL height. The majority of the data, however, are found outside of the surface layer. The exact distribution of data points within the regimes does depend on which diagnostic for SBL height we use. For example, a large fraction of data falls within the neutral upper layer when using *h*_SBL_, while for *h*_anis_ there are no data within that regime. Still, the majority of the data points are located within the *z*‐less regime. The transition between the different SBL regimes, however, is continuous, and the only discontinuity is the large gap between boundary‐layer and above‐boundary‐layer turbulence. Similarly, there is no clear distinction in anisotropy between the different SBL regimes, apart from the stationary turbulence above the SBL, which is more anisotropic (cf. darker colour of points).

Using the scaling diagram, we can now separate the data according to which scaling regime they fall into (surface layer, near‐neutral upper layer, *z*‐less and above the SBL). We then employ the local scaling framework (cf. Nieuwstadt, 1984a; 1984b) to examine how successful the scaling for each regime is and how much the scaled data deviate from the common scaling curves. We compare the scaled data to the flux‐gradient and flux‐variance relations used in literature for flat terrain (Högström, 1988; Stull, 1988) and katabatic flows (Nadeau *et al*., 2013, Grachev *et al*., 2016) as well as numerically integrated scaling relations on a slope developed by Łobocki (2014). Here we need to note that the scaling relations of Łobocki (2014) are not strictly valid for buoyancy‐driven flows and that the scaling relations of Grachev *et al*. (2016) were derived in the turbulent layer above the jet maximum. We examine the scaling relations for non‐dimensional vertical wind shear (*ϕ*_*m*_) following Businger *et al*. (1971) and Dyer (1974):

(16a) $$\phi_{m}=\frac{\kappa \text{z}}{u_{*}}{\left[{\left(\frac{\partial U}{\partial z}\right)}^{2}+{\left(\frac{\partial V}{\partial z}\right)}^{2}\right]}^{1/2}=\left(1+4.7\frac{z}{\Lambda }\right),\;$$

Grachev *et al*. (2016):

(16b) $$\phi_{m\text{\_}\text{Grachev}}=\left(1+4.1\frac{z}{\Lambda }\right),$$

and Högström (1988):

(16c) $$\phi_{m\text{\_}\text{Hogstrom}}=\left(1+6\frac{z}{\Lambda }\right).$$

The scaling relation for the non‐dimensional temperature gradient (*ϕ*_*H*_) following Businger *et al*. (1971) and Dyer (1974):

(17a) $$\phi_{H}=\frac{\kappa \text{z}}{\theta_{*}}\frac{\partial \theta }{\partial z}=\left(0.74+4.7\frac{z}{\Lambda }\right),$$

and Högström (1988):

(17b) $$\phi_{H\text{\_}\text{Hogstrom}}=\left(0.95+7.8\frac{z}{\Lambda }\right).$$

The scaling relations for the standard deviations of velocity component into the mean wind direction (*ϕ*_*u*_) and vertical (*ϕ*_*w*_) velocity following Nadeau *et al*. (2013) and Grachev *et al*. (2016):

(18a) $$\phi_{u\text{\_}\text{Nadeau}}=\frac{\sigma_{u}}{u^{*}}=2.85{\left(1+10.55\frac{z}{\Lambda }\right)}^{1/3},$$

(18a) $$\phi_{u\text{\_}\text{Grachev}}=2.3,$$

(19a) $$\phi_{w\text{\_}\text{Nadeau}}=\frac{\sigma_{w}}{u^{*}}=0.95{\left(1+11.23\frac{z}{\Lambda }\right)}^{1/3},$$

(19b) $$\phi_{w\text{\_}\text{Grachev}}=1.5.$$

Finally, the scaling relation for standard deviation of temperature (*ϕ*_*θ*_) following Sfyri *et al*. (2018):

(20a) $$\phi_{\theta }=\frac{\sigma_{\theta }}{\theta^{*}}=8.7\cdot 10^{-4}{\left(\frac{z}{\Lambda }\right)}^{-1.4}+2.03,$$

and Nadeau *et al*. (2013):

(20b) $$\phi_{\theta \text{\_}\text{Nadeau}}=3.22{\left(1+0.83\frac{z}{\Lambda }\right)}^{-1/3}.$$

Here *κ* is the von Kármán constant, Λ the local Obukhov length, u_*_ is the local friction velocity calculated from both the streamwise and spanwise momentum flux, and θ_*_ is the local temperature scale defined as $\theta_{*}=-\bar{w^{\prime }\theta^{\prime }}/u_{*}$.

**Figure 14:**
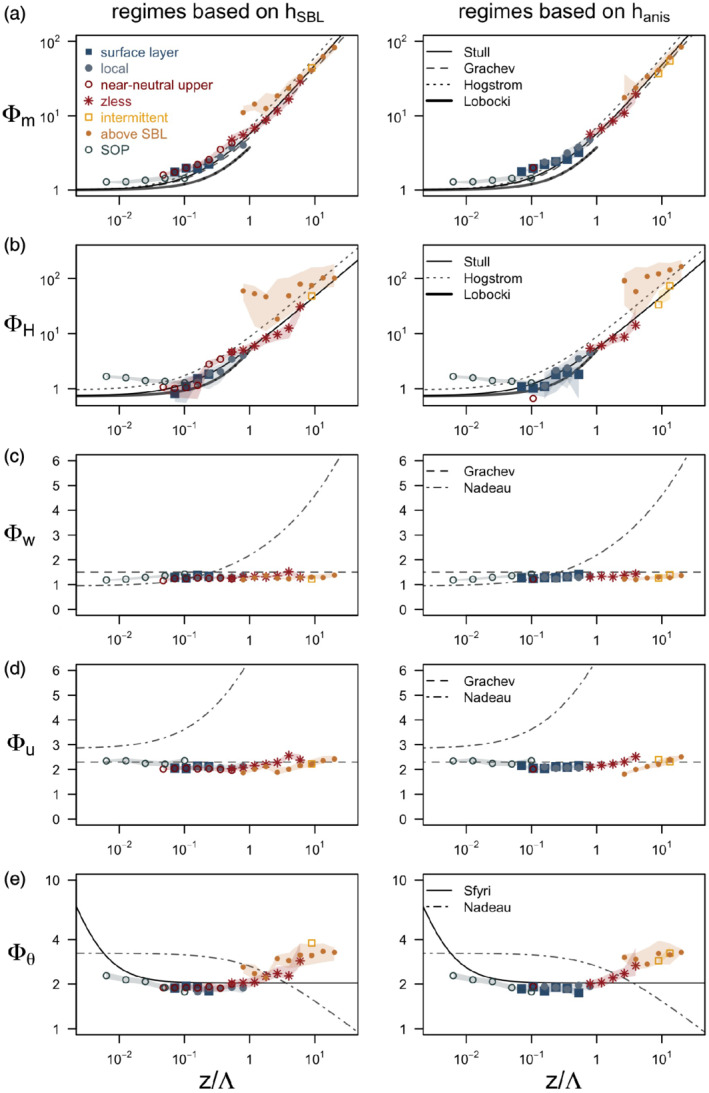
Flux‐gradient and flux‐variance relations for non‐dimensional (a) wind shear (Φ_*m*_), (b) temperature gradient (Φ_*H*_), and non‐dimensional standard deviations of (c) slope‐normal velocity (Φ_*w*_), (d) velocity component into the mean wind direction (Φ_*u*_) and (e) temperature (Φ_*θ*_), for different scaling regimes defined in Figure 13. Regimes defined using *h*_SBL_ as SBL height are shown on the left, while those defined using *h*_anis_ are on the right. Shown are bin averages of 1 min data from all IOPs except IOP5 as well as the SOP that satisfy the stationarity criterion and for which the flux Richardson number is below 0.21. The bin averages are calculated using logarithmic spacing on the *x*‐axis, where points represent medians and the shading corresponds to the interquartile range. The scaling lines correspond to the theoretical curves defined in Equations 16–20 (the name of the first author is indicated in the legend of each figure) [Colour figure can be viewed at wileyonlinelibrary.com]

The scaling relations from different scaling regimes are shown in Figure 14. For comparison, we also add the data from the lowest two measurement levels during the SOP, which we presume to belong within the surface layer. The data are required to fulfill the stationarity criterion and the criterion that *R*_*f*_ is below 0.21 (cf. Section 2.4). The results show several interesting features. First, there is generally a good agreement between the scaled gradients and variances of all SBL scaling regimes with the surface‐layer scaling relations for *z*/Λ = 0.1–1. The exception is the scaled temperature gradient which shows larger deviations. This might be due to comparatively poor performance of analytical formulations for fitting temperature profiles (not shown).

Second, for strongly stable conditions when *z*/Λ ≥ 1 the data from all regimes deviate more strongly from all the examined scaling curves. As expected, this deviation is the largest for the more anisotropic data above the SBL, where the spread is also largest, indicating a general lack of scaling. The data that cause the largest departure from the scaling curve (these include all the data in the intermittency regime) are eliminated by applying the *R*_*f*_ criterion (filtering data with *R*_*f*_ > 0.21) following Grachev *et al*. (2013) (see supplementary Figure S1 for scaling relations without this criterion). The existence of data points from above the detected SBL that nevertheless satisfy the critical *R*_*f*_ criterion (compare Figure 14 and S1) shows that localized Kolmogorov turbulence can develop at higher levels, but its larger scatter and strong departure from the scaling curves, especially for the scaled temperature variables, suggests that this turbulence is decoupled from the surface and does not conform to known scaling relations.

Thirdly, the most surprising result is that both the surface‐layer scaling data from katabatic periods and the data from SOP deviate from the standard scaling curves for *z*/Λ < 0.1. Finally, we can observe that all of the scaled variables generally follow the *z*‐less scaling curves, but strongly depart from the scaling curves of not only Högström (1988), but also Nadeau *et al*. (2013) and Łobocki (2014).

These results, and the fact that the deviation from scaling curves is not strongly dependent on the choice of the diagnostic SBL height, suggest that the difference between the SBL scaling regimes is less important than the stability range over which the scaling is examined. The exception here is of course the turbulence above the SBL. The causes of the deviations from the scaling curves for near‐neutral stratification are outside the scope of this article.

## 6 SUMMARY AND CONCLUSIONS

Relatively deep and persistent katabatic flows develop on a gentle mesoscale slope with a long fetch (of around 30 km) outside the Barringer Meteorite Crater in Arizona. The low slope angle causes the formation of deep flows with jet maxima in the developed stage ranging between 15 and 45 m, and average 5 min speed at *h*_jet_ reaching up to almost 8 m·s^−1^. These deep katabatic flows show a similar near‐surface turbulent structure to shallow katabatic flows developing over steeper slopes (cf. Figure 3). The fluxes (apart from the sensible‐heat flux) are non‐constant with height even below 10 m height, and their magnitudes decrease as *h*_jet_ is reached. Above the jet maximum, on the other hand, no continuous turbulence is maintained, making turbulence profiles markedly different from those of shallow katabatic flows.

While the well‐developed quasi‐isotropic turbulence is caused by shear generation below the katabatic jet maximum, its cessation above *h*_jet_ is a result of the jet maximum being embedded within the inversion (a feature commonly found for mesoscale katabatic flows), where buoyancy suppression of turbulence dominates the shear generation. This height is correlated to the height where *R*_*f*_ is larger than a critical value of 0.21 (Figure 4) but is found even somewhat above it. Still, significant anisotropic kinetic energy and non‐zero streamwise heat flux at sub‐meso scales are found above the weakly stable BL (Figure 6). There is, however, no visible influence of the sub‐meso motions on the small‐scale quasi‐isotropic turbulence below the jet due to the existence of a spectral gap (cf. Figure 6).

Unlike in other studies, *h*_jet_ is not the relevant length‐scale determining the turbulent structure of these deep katabatic flows and is not correlated with the maximum height to which continuous and quasi‐isotropic turbulence is maintained (SBL height). The SBL height itself reaches approximately 43 m at its largest extent, although the exact value is dependent on the definition of the SBL top: somewhat larger if the height where turbulence becomes more anisotropic is used than if height where fluxes become insignificant is used to detect the SBL top (Figure 8). Here anisotropy was shown to be a useful variable in discriminating between well‐developed boundary‐layer turbulence and more anisotropic turbulence above the SBL.

The height of the katabatic SBL was found to depend on the same forcing mechanisms (stability profile, surface values of sensible‐heat flux and friction velocity) as that of the SBL over flat terrain, showing good correlation with the commonly used SBL height formulations (Figure 10), but only when the local near‐surface stability was used instead of the free tropospheric background stratification. Still, despite the low slope angle, the SBL height of the katabatic flow is significantly shallower (up to six times) than it would be over flat terrain for the same forcing, even when accounting for the larger near‐surface stability. The exact role of the slope angle on SBL height is, however, impossible to assess with only one dataset. The length‐scale of the dominant eddy obtained from the MRD co‐spectrum of the sensible‐heat flux, which is shown to be constant with height in katabatic flow, offers another measure of the SBL height, which could allow estimating the katabatic turbulent depth from single‐level surface turbulence measurements.

Estimates of the SBL heights offer the possibility of assessing the different SBL scaling regimes. The results of a number of flux‐gradient and flux‐variance relations from the individual scaling regimes show that the assignment into a scaling regime itself is not relevant as long as turbulence is measured within the SBL. Indeed, the scaling relations from all scaling regimes generally follow the accepted flat‐terrain *z*‐less scaling curves in the stability range of *z*/Λ = 0.1–1, but depart largely from them for very stable and near‐neutral stratification. The deviations are the largest for scaled temperature gradients and temperature variances. On the very stable side, these deviations are due to the inclusion of non‐Kolmogorov turbulence and can be eliminated if *R*_*f*_ is required to be below 0.21. The deviations from the scaled temperature curves have yet to be explained.

The scaling regimes suggest that surface‐layer turbulence can develop at the lowest measurement levels in cases where the SBL is deep. Without a measurement level lower than 3 m, however, it is impossible to confirm if the requirement that the momentum fluxes be constant with height is also met.

In summary, the deep mesoscale katabatic flows outside Arizona's Meteor Crater show both similarities and differences to shallow katabatic flows on steeper slopes and to flat‐terrain SBLs. The fact that they are driven by a slope angle as small as 1° stresses the importance of appropriately including their effect in numerical models, particularly in terms of SBL depth and scaling.

## Supporting information


**Figure S1** shows the flux‐gradient and flux‐variance relations for different scaling regimes of the katabatic flow outside of the Meteor Crater in Arizona, for data that are stationary only and no Richardson number criterion is applied (cf. Figure 14).
