# Supplementary material for: On the turbulence structure of deep katabatic flows on a gentle mesoscale slope
Source: Q J R Meteorol Soc. 2020 Jan 23;146(728):1206–31. doi: 10.1002/qj.3734 (PMC7654318; doi:10.1002/qj.3734)
Supplement: Supplementary file 1 — Figure S1 shows the flux‐gradient and flux‐variance relations for different scaling regimes of the katabatic flow outside of the Meteor Crater in Arizona, for data that are stationary only and no Richardson number criterion is applied (cf. Figure 14). [file QJ-146-1206-s001.docx]

**SUPPLEMENTARY FIGURE**


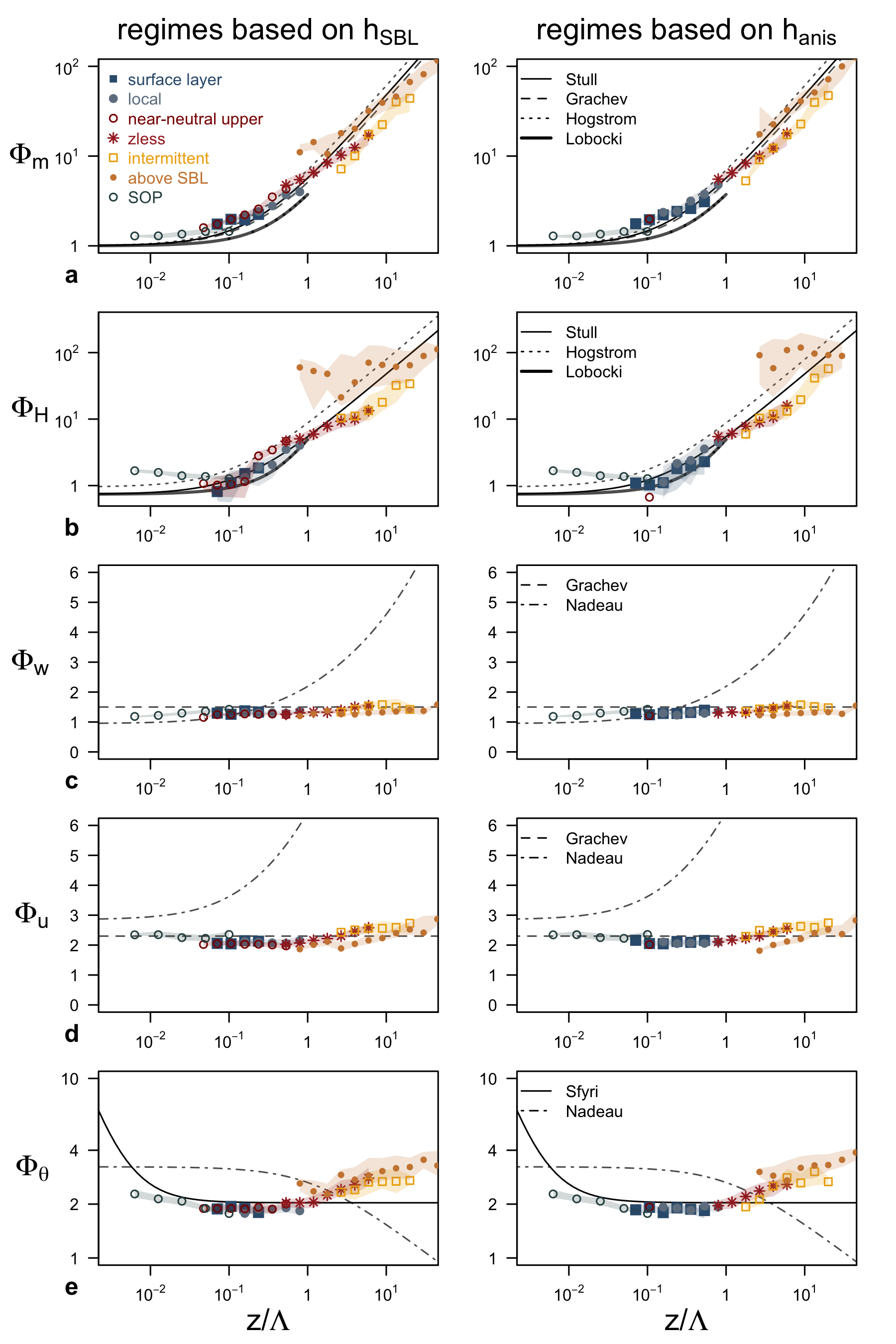


**Figure S1.**

Flux-gradient and flux-variance relations for non-dimensional (a) wind shear (Φ_m_), (b) temperature gradient (Φ_H_), and non-dimensional standard deviations of (c) slope-normal velocity (Φ_w_), (d) along-slope velocity (Φ_u_) and (e) temperature (Φ_θ_), for different scaling regimes defined in **Fig 13.** Regimes defined using *h_SBL_* as SBL height are shown on the left, while those defined using *h_anis_* are on the right. Shown are bin averages of 1 min data from all IOPs except IOP5 as well as the SOP that satisfy the stationarity criterion only while the flux Richardson number is not limited. The bin averages are calculated using logarithmic spacing on the x axis, where points represent medians and the shading corresponds to the interquartile range. The scaling lines correspond to the theoretical curves defined in Eqns. 16-20 (the name of the first author is indicated in the legend of each figure).
